# Self-Nano-Emulsifying Drug-Delivery Systems: From the Development to the Current Applications and Challenges in Oral Drug Delivery

**DOI:** 10.3390/pharmaceutics12121194

**Published:** 2020-12-09

**Authors:** Aristote B. Buya, Ana Beloqui, Patrick B. Memvanga, Véronique Préat

**Affiliations:** 1 Advanced Drug Delivery and Biomaterials, Louvain Drug Research Institute, Université Catholique de Louvain, Avenue Mounier 73, B1.73.12, 1200 Brussels, Belgium; aristote.buya@uclouvain.be (A.B.B.); ana.beloqui@uclouvain.be (A.B.); 2 Pharmaceutics and Phytopharmaceutical Drug Development Research Group, Faculty of Pharmaceutical Sciences, University of Kinshasa, Kinshasa XI BP 212, Democratic Republic of the Congo; patrick.memvanga@unikin.ac.cd

**Keywords:** oral bio-availability, self-nano-emulsifying drug-delivery systems (SNEDDSs), oral delivery, solubilization, food effect

## Abstract

Approximately one third of newly discovered drug molecules show insufficient water solubility and therefore low oral bio-availability. Self-nano-emulsifying drug-delivery systems (SNEDDSs) are one of the emerging strategies developed to tackle the issues associated with their oral delivery. SNEDDSs are composed of an oil phase, surfactant, and cosurfactant or cosolvent. SNEDDSs characteristics, their ability to dissolve a drug, and in vivo considerations are determinant factors in the choice of SNEDDSs excipients. A SNEDDS formulation can be optimized through phase diagram approach or statistical design of experiments. The characterization of SNEDDSs includes multiple orthogonal methods required to fully control SNEDDS manufacture, stability, and biological fate. Encapsulating a drug in SNEDDSs can lead to increased solubilization, stability in the gastro-intestinal tract, and absorption, resulting in enhanced bio-availability. The transformation of liquid SNEDDSs into solid dosage forms has been shown to increase the stability and patient compliance. Supersaturated, mucus-permeating, and targeted SNEDDSs can be developed to increase efficacy and patient compliance. Self-emulsification approach has been successful in oral drug delivery. The present review gives an insight of SNEDDSs for the oral administration of both lipophilic and hydrophilic compounds from the experimental bench to marketed products.

## 1. Introduction

The oral administration route remains the best choice for drug delivery owing to its safety, patient compliance, and capacity for self-administration. In addition to being the most convenient route of administration, oral delivery has been limited owing to the numerous barriers present at the gastro-intestinal (GI) tract [1,2]. The solubilization of the drug within the GI tract is a mandatory for the drug absorption, as insufficient drug dissolution may lead to incomplete absorption, low bio-availability, and high variability following oral administration [3]. The oral delivery of drugs may also be associated with precipitation, food and drug interactions, susceptibility to degradation, and first-pass metabolism, leading to low oral bio-availability. According to the BCS (Biopharmaceutical Classification System), most of the drugs discovered thus far are classified into class II (low solubility, high permeability) and class IV (low solubility, low permeability). Following their oral administration, these compounds exhibited low oral bio-availability due to their low solubility or membrane permeability. Therefore, there is an urgent need to develop new drug carriers for their oral delivery.

The fact that the oral absorption of poor water-soluble drugs could be improved once given with food rich in lipids has brought the use of lipids-based formulations as means to improve the drug solubility and absorption following the oral administration [4]. Lipid-based formulations are considered to be a promising approach to enhance the water solubility and oral absorption of lipophilic drugs. The main goal of these formulations is to maintain the drugs in solution within the GI tract [5]. Among the wide number of lipid-based drug-delivery systems, self-nano-emulsifying drug-delivery systems (SNEDDSs) are one of the most investigated in oral drug delivery. 

**Figure 1:**
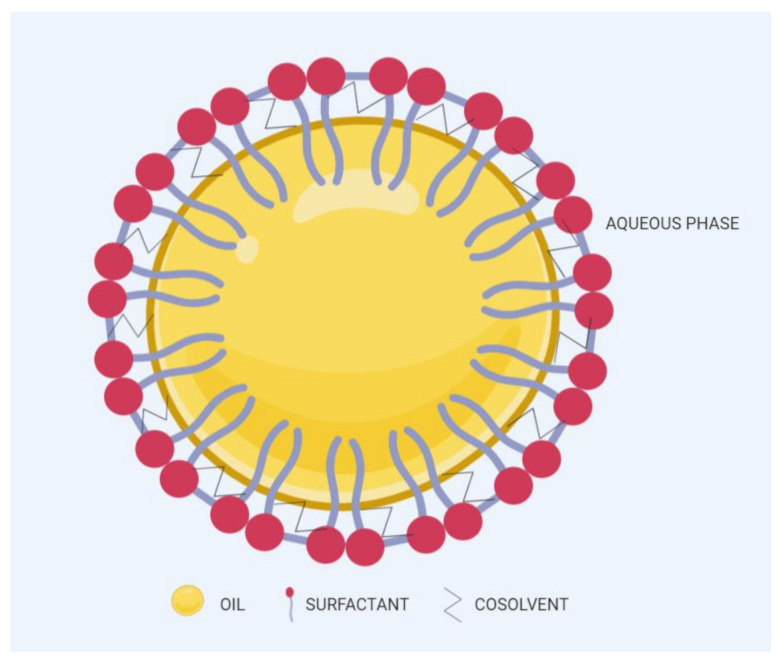
Typical structure of SNEDDSs after aqueous dispersion.

SNEDDSs have been described as a blend of oils, surfactants, and cosurfactants or cosolvents [6]. Following aqueous dispersion and mild agitation (such in GI tract), SNEDDSs spontaneously form fine oil-in-water nano-emulsions with droplet size of 200 nm or below [7], as shown in Figure 1. The spontaneous emulsification takes place when the entropy change favoring dispersion exceeds the energy required to increase the surface area of the dispersion [8,9]. SNEDDSs have shown immense potential in overcoming limitations related to the oral administration of several compounds. Such limitations include low solubility in the GI tract, inconsistent dissolution, enzymatic degradation, and erratic intestinal absorption. Surfactants and lipid components used in SNEDDSs can cooperate to enhance the GI absorption drugs. Furthermore, these components can be modified easily according to the need to make SNEDDSs feasible for both hydrophilic and hydrophobic drugs. Recent studies have shown that SNEDDSs could be effective oral drug carriers of peptides and proteins by preventing their GI degradation and improving their intestinal membrane permeability [10,11,12].

In comparison to other lipid nanocarriers such as nanostructured lipid carriers (NLCs), solid lipid nanoparticles (SLNs) or liposomes or solid dispersions, SNEDDSs can be easily scaled up by mixing components with conventional equipment and then including the mixture in solid dosage form, i.e., capsule or tablet. Furthermore, drug-delivery-system-related issues such as a tendency to aggregate during the storage or to release the drug are not relevant to SNEDDSs, as fine dispersion are directly produced in the GI tract [13]. Therefore, SNEDDSs display better pharmaceutical properties for enhancing solubility and oral bio-availability [7,13]. More recently, however, the development of marketed SEDDSs formulations, such as Norvir^®^ (ritonavir), Sandimmune^®^ (cyclosporine), Fortavase^®^ (saquinavir) and Neoral^®^ (cyclosporine), has stimulated a growing interest in the use of SNEDDSs to improve the drug solubility and oral bio-availability.

To date, there are several studies that focus on SNEDDSs use for the oral delivery of lipophilic compounds, yet relatively few that introduce the potential of SNEDDSs for improving the oral delivery of hydrophilic macromolecules.

**Figure 2:**
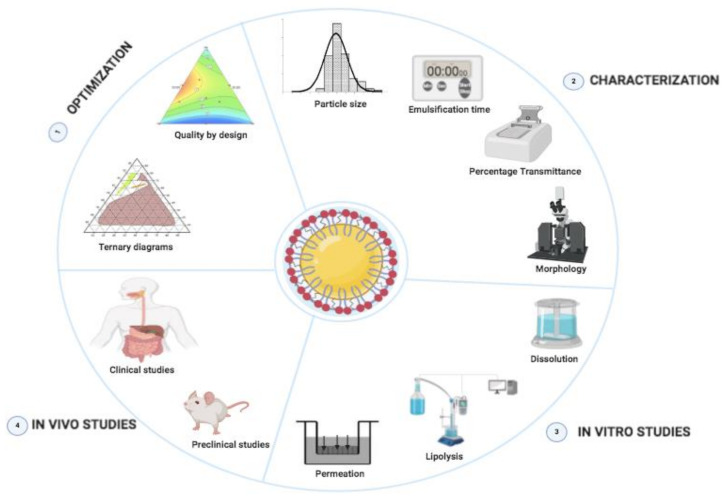
Overview of the design of SNEDDSs formulations.

This paper offers a comprehensive overview of SNEDDSs development, characterization and in vitro/in vivo evaluation (Figure 2). We focus on SNEDDSs use for the oral delivery of both lipophilic and hydrophilic drugs, with special emphasis on the primary mechanisms by which components used to prepare SNEDDSs can improve the drug solubility, stability, and bio-availability after oral administration. Additionally, we discuss some advancements and promising techniques, such as solidification techniques for transforming liquid SNEDDSs into solid SNEDDSs formulations, as well as supersaturated SNEDDSs to enhance the drug-loading capacity. Lastly, we highlighted the most important challenges ahead related to SNEDDSs formulations.

## 2. General Components of SNEDDSs and Their Role in Formulation Performance

To enable differentiation among various lipid-based carriers, Pouton et al. [7] introduced the lipid formulation classification system (LFCS). According to LFCS, SNEDDSs belong to class III compositions, which are composed of oils and water-soluble surface-active agents (surfactants and cosurfactants) and may also include cosolvents. Successful formulation of a SNEDDS requires attention when selecting formulation ingredients. Preformulation studies (e.g., solubility, emulsification efficiency) should be carried out to guide the right selection of SNEDDSs ingredients. 

The general components used for SNEDDSs formulation are summarized below.

### 2.1. Oil Phase

Generally, medium- and long-chain triglycerides (TG) containing oils presenting varying degrees of saturation are used to formulate SNEDDSs. The oil with maximum ability to solubilize a specific a drug is usually selected due to its key influence in both formulation-loading capacity and drug absorption [14]. However, one exception to this general rule was reported by Larsen et al. [15], who demonstrated that SNEDDS containing an oil with the lowest solubilization capacity exhibited the highest drug absorption, indicating that the high solubilization in an oil is not always the best indicator of better in vivo performance.

Natural edible oils (i.e., castor oil, soybean oil, coconut oil, etc.) remain the logical and desired oil ingredients, but they exhibit relatively low drug-loading capacity and poor emulsification efficiency [16]. Modified medium-chain triglycerides (MCTs) and long-chain triglycerides (LCTs) are mostly employed to enhance the drug solubility in the formulation and are presented in Table 1.

**Figure 3:**
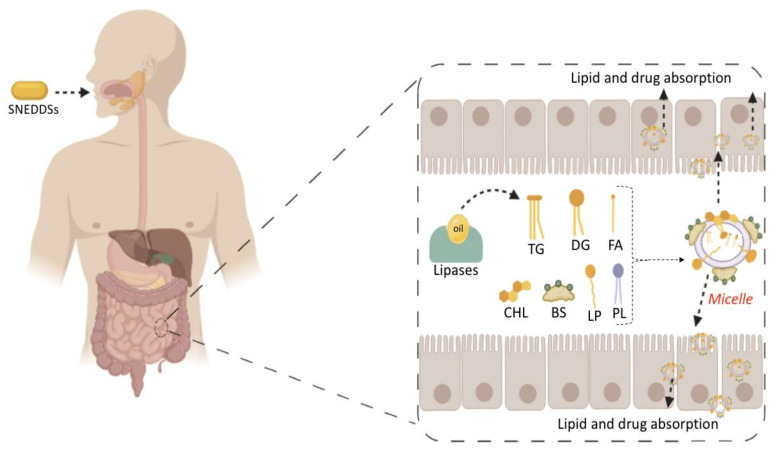
Lipid digestion and drug solubilization process in the small intestine. Abbreviation: triglycerides (TG), di-glycerides (DG), monoglycerides (MG), fatty acids (FA), cholesterol (CHL), bile salts (BS), lipoproteins (LP), phospholipids (PL).

MCTs are predominantly composed of triglycerides with lipid chain lengths ranged from lipid chain lengths ranging from C_8_ to C_10_ (i.e., Capryol^®^ 90, Captex^®^ 300, Labrafac^®^ CC), whereas LCTs consist of TG with lipid chain lengths greater than C_10_ (e.g., Maisine^®^-35, Lauroglycol^®^ 90, Peceol^®^) [18]. After oral administration of these lipids, gastric, and pancreatic lipases break down TG into diglyceride, monoglyceride, and fatty acids. Once within the small intestine, those products stimulate the release of endogenous biliary lipids from the gall bladder, including bile salt, lipoprotein, phospholipid, and cholesterol, which enhance the solubilization and absorption ability of the intestinal tract via the formation of micelles (Figure 3) [19,20,21].

MCTs are preferred because of their better solubilizing ability and self-emulsification capacity [22]. C10 remains the only enhancer that has been used clinically in the intestine for oral drug delivery [23]. MCTs can increase the drug transport through the portal vein, but they have a limited capacity to enhance the lymphatic transport of the drugs [24,25]. Conversely, LCTs are directly encapsulated into chylomicrons, before their passage into the lymphatic system, bypassing the hepatic first-pass metabolism [4,25,26]. LCTs increase the transport of drugs through lymph vessel; however, sometimes, they are difficult to emulsify [27]. Thus, a mixture of MCTs and LCTs can be considered to meet optimum properties and improve pharmacokinetics.

### 2.2. Surfactants

The second obligatory components in SNEDDSs are surfactants. Due to their amphiphilic properties, surfactants are found at the oil–water interface and help in the stabilization of the nano-emulsion by reducing the surface tension. Generally, surfactants are classified based on their charge and hydrophilic-lipophilic balance (HLB) value. Regarding their charge, surfactants are categorized as ionic (anionic, cationic, and zwitterionic) and non-ionic surfactants. As compared with ionic surfactants, non-ionic surfactants are generally used because of their lower toxicity and ability to stabilize emulsion over a wider range of nano-emulsion pH and ionic strength [28]. Regarding their HLB value, surfactants can be classified as lipophilic (HLB < 10) or hydrophilic (HLB > 10) surfactants. The non-ionic surfactants with HLB > 12 are the most recommended, as they enable a spontaneous nano-emulsification with particle sizes less than 200 nm after aqueous dispersion. 

The emulsification ability of a surfactant, its HLB value and the maximum solubility of the drug are three important factors to keep in mind when selecting surfactant in SNEDDSs. Furthermore, the concentration of surfactant has been demonstrated to affect the emulsion particle size. Increasing the amount of surfactant can reduce the emulsion particle size due to the surface tension lowering property of the surfactant at the oil and water interface that reduces the free energy for emulsification [14]. However, in some cases, an increase in surfactant amount results in higher particle size, due to the excess penetration of water into the lipid droplet which cause massive disruption of the oil–water interfacial and relaxation of high polydisperse nano-emulsion droplets [29,30]. Other than fine globule formation, many non-ionic surfactants, such Tween^®^ 80 and Cremophor^®^ EL, possess the ability to increase membrane fluidity [31] and to inhibit efflux transporters [32,33], which are contributing factors in enhancing the drug bio-availability.

The surfactant acceptability for the oral delivery and its regulatory status (e.g., GRAS—generally regarded as safe) should also be taken into consideration during the selection. Table 1 presents common non-ionic surfactants along with their acceptability. It should be noted that surfactant molecules are not always innocuous, they can exhibit structure or concentration-dependent toxicity [17]. Some of them might cause irritation the GI epithelium following oral administration. Thus, the amount of surfactant in SNEDDSs must be maintained at a low level as much as possible.

### 2.3. Cosurfactants/Cosolvents

A single surfactant is rarely able to provide low interfacial tension; therefore, the addition of another surfactant (cosurfactant) or cosolvent usually is necessary. They can synergically cooperate with surfactants to enhance the drug solubility and surfactant dispersibility in the oil, thus promoting nano-emulsion stability and homogeneity [34]. The use cosurfactants or cosolvents can reduce the local irritancy of the surfactant and dose variability of the formulation by improving interfacial fluidity [35]. The weight ratio of surfactant/cosurfactant or cosolvent has also been reported to have an important impact on size distribution and the extent of nano-emulsion area [36,37]. Commonly used cosolvents include propylene glycol, ethanol, poly (ethylene glycol) (PEG) and other newer cosolvents, such as Transcutol^®^ HP [38,39], which are presented in Table 1. 

However, while cosolvents can improve drug solubilization in the formulation, their amount should be kept at minimal level because of their polarity. Cosolvent readily migrate toward the water phase following aqueous dispersion, leading to drug precipitation [40]. Furthermore, alcohols and other volatile cosolvents can evaporate into shells of capsules, resulting in drug precipitation [41].

In the SNEDDS formulation, apart from previously presented components, other ingredients such antioxidants, viscosity enhancers and ingredients for modified drug release can be used [42,43,44,45].

## 3. Optimization of SNEDDSs Formulations

After selecting potential components of SNEDDSs, optimization studies are performed to obtain the optimum amounts of oily phase, surfactants, and cosolvents that might yield spontaneous nano-emulsion [46]. Ternary phase diagrams are largely employed to identify the emulsification area for selected components. In ternary diagrams, the ratio of one component varies while the concentrations of the other two are fixed. The emulsification area is identified visually or by measuring the particle size of the emulsion/nano-emulsion resulting after aqueous dispersion. All the SNEDDSs composition from the emulsification area yield spontaneous nano-emulsions, with globule sizes less than 200 nm after aqueous dispersion [47]. In some cases, the drugs can influence the emulsification region. Date et al. [48] demonstrated that cefpodoxime proxetil could significantly reduce the emulsification region in the ternary phase diagram. 

**Figure 4:**
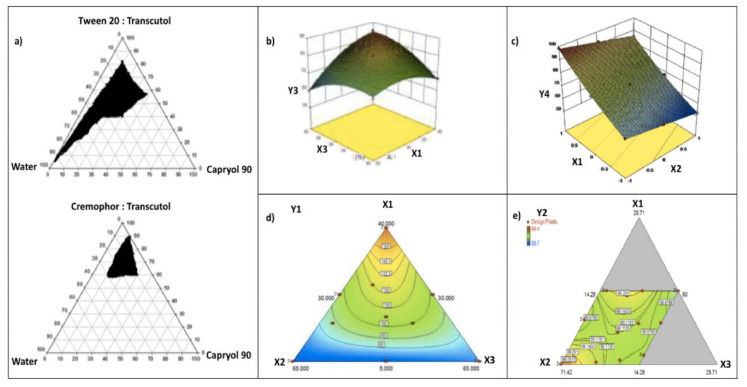
Optimization of SNEDDSs (**a**) ternary diagrams from [49], (**b**) Box–Benkhen design from [58]. Drug: polypeptide-k, Factors Oleoyl polyoxyl-6 glycerides (oil, X1), Tween^®^ 80 (surfactant, X2), diethylene glycol monoethyl ether (cosolvent, X3); responses: percentage drug loading (Y3), (**c**) central composite design from [53]. Drug: Bosentan, Factors: Capmul^®^ and Labrasol^®^ (surfactants, X1), MCM (oil, X2), and PEG 600 (cosolvent, X3); responses: percentage drug release in 15 min (Y4), (**d**) simplex lattice design from [61]. Drug: pentagamavunon-0, Factors: oil (oleic acid, X1), surfactants (Tween^®^ 20 and Labrasol^®^, X2), cosolvent (PEG 400, X3); response: particle size (Y1) (**e**) D-optimal design from [56]. Drug: cardamom essential oil, Factors: coconut oil (X1), Tween^®^ 80 (X2) and PEG 400 (X3); response: transmittance percentage (Y2).

Khattab et al. [49] developed SNEDDSs to enhance aliskiren hemi-fumarate oral absorption. Capryol^®^ 90 (oily phase), Cremophor^®^ RH and Tween^®^ 20 (surfactants) and Transcutol^®^ HP (cosurfactant) were selected from the solubility study. The formulations were further optimized using a pseudo-ternary phase diagram in which an area of emulsification was identified (Figure 4a). The region of nano-emulsification was defined as the region where homogenous and clear systems were obtained after aqueous dispersion. A large nano-emulsion area indicates better emulsification efficiency of the surfactant toward oil. For Tween^®^ 20/Transcutol^®^ HP systems, they showed that increasing the Tween^®^ 20 to Transcutol^®^ HP ratio increased the nano-emulsion area, which was explained by the increase in surfactant adsorption at the emulsion interface leading to decreases in surface tension and formulation droplet sizes. For Cremophor^®^ RH 40/Transcutol^®^ HP systems, the opposite was noted. They observed that increasing the Cremophor^®^ RH 40 to Transcutol^®^ HP ratio resulted in a notable decrease in the nano-emulsion region. The fact was explained by the high viscosity of Cremophor^®^ RH 40, preventing a rapid breakage of the oil–water interface, and thus decreasing the area of nano-emulsion. The authors concluded that Tween^®^ 20 could better emulsify Capryol^®^ 90 compared to Cremophor^®^ RH 40. The final SNEDDS consisted of Capryol^®^ 90 (oil), Tween^®^ 20 (surfactant), Transcutol^®^ HP (cosolvent) and improved the oral bio-availability of aliskiren hemi-fumarate in rats compared to drug solution.

In addition to a ternary phase diagram, SNEDDSs optimization can also be done with numerous types of statistical experimental design, such as Box–Benkhen design [50,51,52], central composite design [53], simplex lattice design [54], full-factorial design [55], and D-optimal design [56]. 

Box–Benkhen design is a response surface design based on three levels (−1, 0, +1) which provides an appropriate model for the quadratic behavior of factors [57]. The number of runs (*N*) needed to develop Box–Benkhen design is given as *N* = 2k(k − 1) + C_0_, (where k and C_0_ are the numbers of independent variables and central points, respectively). Garg et al. [58] formulated SNEDDSs of polypeptide-k that were optimized by Box–Benkhen design (Figure 4b). Seventeen runs were performed to study the impact of SNEDDS factors on the selected responses (dependent variables). From the study, a decrease of size (Y1) was observed at a higher level of surfactant (Tween^®^ 80, X2), while size increased at higher levels of oil (oleoyl polyoxyl-6 glycerides, X1) and cosolvent (diethylene glycol monoethyl ether, X3). The drug loading (Y3) increased with the increases in X1, X2, and X3 ratios, as shown in Figure 4b. Furthermore, more negative values of zeta potential (Y4) were observed when the concentration of oil (oleoyl polyoxyl-6 glycerides, X1) increased. The optimized SNEDDS showed values of droplet size (Y1), 32 nm, drug loading (Y3), 73%; and zeta potential (Y4), −15.6 mV, and enhanced the oral bio-availability of polypeptide-k in rats. 

Central composite designs are the most largely employed response surface designs. They are fractional factorial or factorial designs containing center points, along with a group of axial points which enable the estimations of curvature [59]. The experimental design must have at least three levels of each factor another to establish the coefficients of a polynomial with quadratic ter. A central composite design requires 2^k^ + 2k + n_c_ experiments, where k and n_c_ are the numbers of factors and central points, respectively. 

Panigrahi et al. [53] optimized by central composite design bosentan loaded SNEDDSs composed of Capmul^®^ and Labrasol^®^ (surfactants, X1), MCM (oil, X2), and PEG 600 (cosolvent, X3). Preliminary Taguchi design studies revealed surfactant and oil as important factors in SNEDDSs that were further screened and optimized by central composite design. For particle size (Y1), it was observed that at a medium to high concentration of surfactant, Y1 increased only when the amount of oil was reduced. Furthermore, particle size (Y1) was increasing with the gradient declination of surfactant amount. For emulsification time (Y2), it was observed that the gradient increase in surfactant amount reduced Y2. It also signified that an increase in oil amount will increase the Y2. In the case of percentage drug release in 15 min (Y3), it was observed that at a low level of oil, Y3 was high only when the amount of surfactant was higher. Y3 was decreasing on the gradient declination of surfactant amount (Figure 4c). The optimized SNEDDS revealed values of particle size (Y1), emulsification time (Y2) and percentage drug release in 15 min(Y3) as 62.5 nm, 12 s, and 98.5%, respectively, and improved bosentan oral bio-availability as compared to pure drug in rabbits.

Simplex lattice design is defined as a space-filling design which creates a triangular grid of experiments (runs). In this design, the fractions of excipients that make up any composition must add to unity; hence, a regular simplex represents factor space. Mixture points are evaluated in accordance with a lattice arrangement, and a simplified polynomial function is used to represent dependent variables [60]. This function represents how the components affect the response. This design offers an effective tool for investigating the properties of blends over wide ranges of composition, especially for mixtures of four or more components. 

With the aim of improving the dissolution rate of pentagamavunon, Astuti et al. designed SNEDDSs formulations that were optimized using simplex lattice design. The factors were the concentrations of oil (oleic acid, X1), surfactants (Tween^®^ 20 and Labrasol^®^, X2), and cosolvent (PEG 400, X3). Particle size (Y1) increased when the amounts of oil (X1), surfactants (X2), and cosolvent (X3) increased (Figure 4d). Moreover, oil concentration had the highest effect on particle size, while the effects of surfactants and cosolvent were more limited. For the drug solubility in the formulations (Y3), the main effect shows a positive coefficient, following the order: cosolvent > surfactants > oil. In addition, the authors showed that the most significant antagonistic interactive effect was X1X2X3; thus, the effect of the three factors together was less than the sum of the three factors taken independently of each other, while the most significant synergistic interaction effect was X1X2. The optimum SNEDDSs consists of 18.6% oleic acid, 51.4% Tween^®^ 20: Labrasol^®^ (1:1) and 30% PEG 400 and showed a size of 75 nm (Y1) and drug solubility of 31.80 mg/mL (Y3) [61].

Full-factorial design is composed of two or more independent variables interacting each other at different levels. This design is used to study the main effects and interactions of independent variables on dependent variables. The number of runs needed to study n independent variables at 2-levels is 2^n^. The full-factorial design is particularly useful in the early stage of the experimental work, especially when the number of independent variables is ≤4 [62].

Karamanidou et al. [63] formulated SNEDDSs for the successful oral delivery of insulin. The authors applied a 3^3^ full-factorial design for selecting the quantities of the components (oil, surfactant and cosurfactant/cosolvent) to be used for each composition. The optimum SNEDDSs were composed of Lauroglycol^®^ FCC as the oily phase, Cremophor^®^ EL as the surfactant, and Transcutol^®^ P or Labrafil^®^ M 1944 CS as the cosurfactant. The systems were characterized by average droplet sizes of 30–45 nm and percentages of insulin loading between 0.27 and 1.12%. They demonstrated that insulin-phospholipid (dimyristoyl phosphatidylglycerol) encapsulation into SNEDDSs improved enzymatic stability of the formulations and a sustained release of insulin from the formulations was observed. The SNEDDSs were innocuous up to concentrations of 2 mg/mL and improved insulin permeability. 

D-optimal design is among designs generated by a computer algorithm. This design should be applied when classical experimental designs cannot be used. Unlike classical experimental designs, D-optimal design usually contains no orthogonal matrices, and effect estimates are correlated [64]. D-optimal design is always applicable regardless of the type of mathematical model used or the specified objective of the experiment. It is a straight response surface design based on a selected optimality criterion and the best fitting model (i.e., first order plus interaction, cubic, full quadratic, etc.) [65,66].

Ujilestari et al. formulated and characterized SNEDDSs of cardamom (Amomum compactum) essential oil. The SNEDDSs formulations were optimized by D-optimal design by varying amounts of coconut oil (X1), Tween^®^ 80 (X2) and PEG 400 (X3). Emulsification time (Y1) and transmittance percentage (Y2) were chosen as response variable for the optimization. They observed a significant (*p* < 0.05) relationship between the factors (X1, X2, X3) and the emulsification time (Y1), while no significant (*p* > 0.05) relationship was observed between the factors and the transmittance percentage (Y2) (Figure 4e). The optimized SNEDDS was composed of 10% cardamom essential oil, 10% coconut oil (X1), 65.7% Tween^®^ 80 (X2), and 14.3% PEG 400 (X3). The SNEDDS exhibited an emulsification time of 46.38 s, 99.37% of transmittance percentage, a viscosity of 187.5 mPa, a particle size of 13.97 nm, and zeta potentials ranging from 28.8 to 45.9 mV. The studies demonstrated that the SNEDDSs had enhanced water solubility and stability of cardamom essential oil [56]. 

Compared with ternary phase diagrams, the key advantage of these statistical experimental designs is that they can minimize expenditure in terms of time, resources, and developmental efforts. Moreover, the simultaneous influence of factors (oil, surfactant and cosolvent) on the SNEDDS’ characteristics (i.e., droplet size, PDI, time of emulsification, etc.) can be studied.

## 4. Physico-Chemical Characterization of SNEDDSs Formulation 

It is always important to evaluate the final SNEDDSs for several parameters. The general techniques and methods that have been employed for SNEDDSs characterization are summarized below (Table 2).

### 4.1. Particle Size

The droplet size of a SNEDDS is often measured after aqueous dispersion via dynamic light scattering (DLS) [67]. The availability of DLS made it a popular technique for droplet size determination; however, the measure can be biased in the presence of large aggregates which scatter more than the nanoparticles, especially at low scattering angles [68,69]. To overcome this limitation, fluorescence correlation spectroscopy (FCS) and Taylor dispersion analysis (TDA) can be used as complementary techniques. In FCS, the fluorescence fluctuations from a fluorescent probe which diffuses in and out of a tiny observation volume is measured [70]. Its high sensitivity allows it to work in dilute solutions; however, FCS applications for larger-sized particles (i.e., emulsion) are still limited, probably owing to the difficulty involved in measuring particle sizes larger than 1/10th of the observation volume’s size [71,72]. Conversely, as a microcapillary-based flow method, TDA allows the characterization of particle size and the stability of small compounds in solution, even for complex composition [73]. TDA quantifies the broadening of the peaks of a specific molecule plug in a Poiseuille laminar flow to determine the molecular diffusion coefficient and subsequently, the hydrodynamic radius [74]. TDA is advantageous as it is less affected by the presence of large-particle aggregates or the sample viscosity; hence, the solutions can be run without any filtration or dilution [75]. However, it usually requires a lipophilic marker which travels in the droplet or micelle [76,77]. The Taylorgrams are plotted as optical density versus time, and the hydrodynamic radius are generated from the molecular diffusion coefficient [73,74]. Chamieh et al. [75] used TDA coupled with a fluorescence detector for the particle size characterization of Labrasol^®^. The particle size characterization was performed at two different temperatures (25 °C and 37 °C) and increasing concentration (from 1 to 70 g·L^−1^). The authors showed that when combined, DLS and TDA allowed determining the proportion and coacervates size in the dispersion as well as the PDI of the sample.

Size characterization is one of the most essential examinations for SNEDDSs development since the size of the particles can directedly affect not only the in vitro tested characteristics (i.e., dissolution, stability) but also the in vivo performance of a SNEDDS. (i.e., drug absorption) [78,79]. The literature reported that smaller particle size has a positive effect on the oral bio-availability of a drug encapsulated into SNEDDSs [80,81]. The plausible explanation for the improved oral bio-availability could be that the smaller the particle size, the larger interfacial area, which improves the drug solubilization and permeability. However, it is not a general rule that a smaller globule size of dispersion will always lead to higher oral absorption. Yap et al. [82] compared the oral bio-availability of tocotrienols from two SEDDSs, the first one yields a large emulsion that readily lipolyzed (E1), while the second produced a smaller emulsion with negligible digestion (E2).

Both E1 and E2 showed the same oral bio-availability even though E2 yield dispersion with a smaller particle size. Thus, it appears that droplet size taken together with other SNEDDSs parameters (i.e., susceptibility to lipolysis) have direct impact on the oral absorption of a compound encapsulated into SNEDDSs. However, despite a lack of consistent correlation between emulsion droplet size and oral absorption, generating a smaller dispersion following aqueous dilution or lipolysis is generally necessary since, it is a known fact that these formulations can minimized dose variability after oral ingestion [83,84,85].

### 4.2. Zeta Potential

The zeta potential provides information about the colloidal stability. It is estimated by measuring the electrophoretic mobility of the droplets. The presence of a high zeta potential value (±40 mV) exhibits repulsive electrostatic forces, which reduces the possibility of particle aggregation [86]. The nanoparticle charge can affect the oral absorption of the drug encapsulated into SNEDDSs. Charge-dependent interaction with mucus and cell membrane barriers with respect to absorption enhancement has been reported [87]. The mucus thin layer protects the GI epithelium from xenobiotics and pathogens, but it also acts as a strong barrier for nanoparticles [88]. The mucus gel exhibits negatively charged substructure made of sulfonic and sialic acid, which hinders positively charged nanoparticles from diffusing into deeper mucus regions owing to electrostatic interactions. Accordingly, negatively charged nanoparticles can more easily permeate the mucus gel compared to positively charged nanoparticles. However, the apical side of the intestinal epithelial cells exhibits negative charges related to the mucosal solution in the lumen. Accordingly, nanoparticles with positive charges can interact with the negative charges of the intestinal mucosal and enhance the cellular uptake of the encapsulated molecule [89,90]. In view of this, Salimi et al. [91] developed SEDDSs that can change their zeta potential via a flip-flop mechanism. They synthesized and incorporated into SEDDSs a conjugate compound that carries both an amino group and a phosphate group. Particles exhibited both a negative value of zeta potential during the mucus transport and a positive zeta potential value after enzymatic degradation of the phosphate ester group, resulting in high cell association and uptake.

### 4.3. Emulsification Time Measurement

Th emulsification time can be measured on a USP II dissolution apparatus [22]. The formulation is added to a basket containing water and is maintained at 37 °C under gentle agitation (100 rpm). The emulsification time is recorded as the time required to obtain a clear dispersion [92]. The emulsification time is dependent on the oil/surfactant concentration. A spontaneous emulsification is observed with surfactant concentrations less than 60% (*w*/*w*) because of the quick release of oil droplets by water penetration into the oil–water interface. However, above the surfactant concentration of 60% (*w*/*w*), there is an increase in the time of emulsification due to the high viscosity of the surfactants [21]. A rapid emulsification can contribute to a quick drug release and a subsequently rapid onset of action [93,94].

### 4.4. Transmittance Percentage Measurement

The transmittance percentage is the measurement of optical clarity of the diluted SNEDDSs with water. The transmittance usually described in percentage is the measurement of how much light passes through a sample. It can be assessed by spectroscopy using water as a blank [95,96]. The increase in transmittance can be used to monitor the self-emulsification rate, and the final transmittance percentage is usually correlated with the nanoparticle droplet size [97,98].

### 4.5. Morphology

The morphology of the nano-emulsion droplets can be determined by scanning electron microscopy (SEM) and transmission electron microscopy (TEM)). SEM is based on back-scattered electrons, which informs the droplet morphology. In TEM, electrons are transported through the dispersion to generate the morphology of the droplets and differentiate several chemical molecules with the respect to their density. Recently, cryo-SEM and cryo-TEM have been developed to study the real morphological information of nanoparticles [74].

### 4.6. Viscosity Measurement

Generally liquid SNEDDSs formulations are filled into capsules. Low-viscosity formulations face leakage concerns, whereas overly viscous SNEDDSs are hardly filled into capsules due to flowability problems [99]. Generally, a viscosity ranging between 0.1–1.0 Pa at 25 °C implies that the formulated SNEDDSs can easily be filled into capsules by liquid filling equipment [100]. The viscosity of SNEDDSs is determined with viscometers.

### 4.7. Cloud Point Measurement

The cloud point is known as the temperature at which the nano/emulsion is broken. The cloud point is determined to investigate the stability of SNEDDSs in the Gl tract. Formulations are diluted with distilled water and placed in a water bath with gradually increasing temperature. Furthermore, spectrophotometric analyses are carried out to determine the transmittance percentage of the sample. At the cloud point, the decrease in dispersion transmittance percentage from the zero point is noted [101,102]. The cloud point of SNEDDSs should be more than 37 °C; otherwise, absorption of the drug can be interrupted, as cloudy emulsion affects the absorption by the dehydration of components used in SNEDDSs formulations [103].

### 4.8. Thermodynamic Stability Studies

The thermodynamic stability is an indicator of the kinetic stability of a dispersion and is generally used to study the chemical reactions occurring between the components of a dispersion. Poor stability of dispersion can lead to precipitation or phase separation, which could affect drug absorption as well as therapeutic efficacy [104,105]. Generally, centrifugation, heating-cooling, and freeze-thaw cycles are carried out for these studies. Various aspects such as phase separation, turbidity, and particle size are observed during these experiments. Subsequently, stable formulations are selected for further evaluation.

## 5. In Vitro Assessment of SNEDDSs Formulations

The literature reports the potential of SNEDDSs in improving the oral bio-availability of several compounds. It is a known fact that the performance of any SNEDDS depends on a complex interplay between physiological processes in the GI tract. Following oral ingestion, the digestion of SNEDDSs is initiated in the stomach, where digestible excipients (oils and surfactants) are lysed by the action of gastric lipase at the interface. Gastric digestion releases approximatively 15% of fatty acids from lipids. Within the small intestine, pancreatic lipase together with its co-lipase complete the breakdown of dietary glycerides to di-glycerides, monoglycerides, and fatty acids. The presence of exogenous lipids in the small intestine also stimulates secretion of endogenous biliary lipids, including bile salt, phospholipid, and cholesterol from the gall bladder. In the presence of an elevated bile salts concentration, lipid digestion products are subsequently incorporated into a series of colloidal structures, including multilamellar/unilamellar vesicles and bile salt phospholipid mixed micelles [19,20,21]. Together, these vesicles significantly increased the solubilization ability of the small intestine for both lipid digestion products and drugs before their absorption. 

Although this knowledge is useful, the prediction of the in vivo performance of a SNEDDS remains challenging. For this purpose, a series of in vitro models or tests have been developed to simulate main processes related to the absorption of drugs. These processes are usually evaluated in various in vitro models testing dissolution, digestion, and permeation. The in vitro models employed vary depending on their physiological relevance and complexity, ranging from single unit to multi-compartmental models. More elaborate in vitro models evaluate dissolution, digestion, and permeation simultaneously [106,107]. Different in vitro models that have been used to evaluate SNEDDSs are described below.

### 5.1. In Vitro Dissolution

The in vitro dissolution test is routinely employed as an indicator of the likely GI drug dissolution and, consequently, as a tool to predict the rate and extent of absorption for poorly water-soluble drugs. The rate of drug dissolution relies on many factors, including the degree of wetting, the drug solubility in the intestinal contents, medium viscosity, emulsion droplet size and the volume of the intestinal contents [108]. The pH has also a key impact on drug dissolution characteristic. Generally, simulated gastric fluid without enzymes (pH 1.2) and phosphate buffer (pH 6.8–7.4) have been used to test drug dissolution. In general, the in vitro dissolution from a SNEDDS formulation is faster compared with native drug due to the reduction in particle size and the increase in surface area [93,94,109]. 

Eleftheriadis et al. [110] studied the dissolution behavior of SNEDDSs loaded with fenofibrate or itraconazole in comparison with the pure drugs. Dissolution studies were performed using a USP dissolution apparatus II in 900 mL of simulated intestinal fluid at 75 rpm paddle rotation and 37 °C. The results showed that the incorporation of these molecules in SNEDDSs significantly enhanced their dissolution rate. Regarding the pure drugs, only 6.6% of fenofibrate and 1.6% of itraconazole were dissolved in 45 min. Almost 100% of the active contents were dissolved from the SNEDDSs formulations in the same period (*p* > 0.05). At the end of the experiment, the total amounts of pure fenofibrate and itraconazole released were 11% and 4%, respectively. In another example, Abouhussein et al. [111] investigated the in vitro dissolution of rivaroxaban loaded SNEDDSs in comparison with the drug powder. The standard USP II paddle method was used at 37 ± 0.5 °C, and 900 mL of sodium lauryl sulfate (0.6%) in acetate buffer pH 4.5 was employed as the dissolution medium. From the studies, it was found that the two developed SNEDDSs provided significantly higher rates of release (100% and 78% in 5 min, respectively) compared to pure raw drug powder (15%).

However, the use of simple aqueous media to test the dissolution behaviors of poorly water-soluble drugs is often limited by two factors: (1) the poor solubility of the drug (and, therefore, the difficulty to maintain sink conditions), which, when coupled with analytical sensitivity issues such as drug binding to filters can make reproducible in vitro dissolution evaluation difficult, and (2) the lack of similarities between the simple aqueous media and the likely GI tract environment, which reduces the in vivo prediction. In attempt of improving the accuracy of in vivo prediction through in vitro dissolution test, many studies have developed and used biorelevant media that more accurately reflect the solubilization capacity of the GI tract [112,113,114,115]. The compositions of these biorelevant media have been inspired mainly by the likely concentration of endogenous phospholipids and bile salts in the stomach and the proximal part of the small intestine [116,117].

Dressman et al. [118] have studied the dissolution behavior of many lipophilic drugs using various dissolution media [119]. Consistent correlations were found for nonionizable drugs between the type of media and the dissolution profiles of the drugs. For example, the percentage release of danazol in fed state intestinal conditions (FeSSIF media) was three-fold higher compared to fasted state intestinal media (FaSSIF). For molecules with appreciable ionization over the physiological pH range, the situation is complicated by the impact of both ionization and media on the drug solubility.

**Figure 5:**
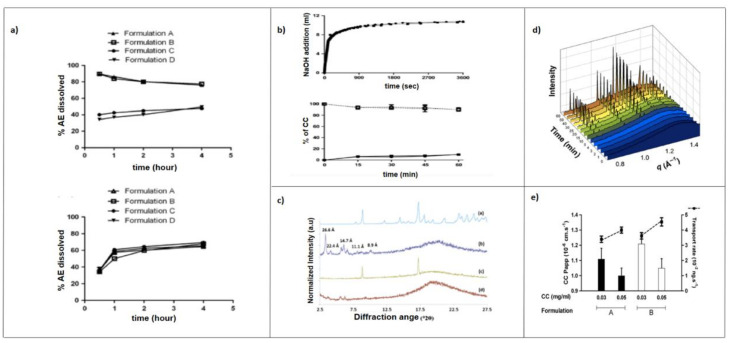
(**a**) Solubilization and stability of beta-Arteether in 0.1 N HCl (pH = 1) (top) and in phosphate buffer (pH = 6.8) (bottom) as a function of time. Each point represents the mean ± SD (*n* = 3). From [41]. (**b**) Quantity of 0.2 M NaOH added to titrate the fatty acids that were released during lipid digestion (top) and the distribution profile of curcumin in the aqueous phase (open shapes and dotted lines) and in the pellet phase (filled shapes and lines) as a function of lipolysis time (bottom). From [40]. (**c**) The X-ray powder diffraction patterns of (a) crystalline, (b) CC pellet, (c) blank pellet spiked with CC and (d) blank pellet from the lipolysis of a SNEDDS formulation. The numbers over the peaks indicate d-spacings. From [40]. (**d**) In situ SAXS profiles during the lipolysis of the MC-SNEDDS formulation containing fenofibrate. Drug precipitation was evident at 4 min after the addition of pancreatic lipase, with the characteristic diffraction peaks for fenofibrate. From [123]. (**e**) Apparent permeability and transport rate of curcumin-loaded SEDDS across Caco-2 monolayers with two different drug concentrations (0.03 and 0.05 mg/mL). From [40].

It was observed for a weak base such as ketoconazole (pKa 6.5, 2.9) that the ionized species at pH 1.2 was much soluble than the unionized at pH 6.5. Furthermore, the percentage of drug dissolved in simulated fasted gastric fluid (FaSSGF) was significantly higher compared to the simulated fasted intestinal fluid (FaSSIF). However, the improved solubilizing capacity of the fed intestine is, at least in part, sufficient to overcome the poor intrinsic solubility of the unionized ketoconazole and the amount of ketoconazole dissolved under fasted gastric state is not notably different from that dissolved under simulated fed state intestinal [120,121]. Memvanga and Préat [41] developed SEDDSs composed of groundnut or sesame oil, Maisine^®^ 35-1, Tween^®^ 80 or Cremophor^®^ EL, and absolute ethanol for the oral delivery of β-Arteether. The in vitro dissolution test using gastric (HCl 0.1 N) and intestinal (phosphate buffer pH 6.8) media showed an increase in drug solubilization over time (Figure 5a). Mendes et al. [122] evaluated the dissolution of hydrochlorothiazide from two SNEDDSs and pure drug. Studies were performed using USP apparatus III containing 200 mL of FaSSGF pH 1.6 or FaSSIF pH 6.5, both at 37 ± 0.5 °C as dissolution media. In the first step of the assay, the dissolution was performed in FaSSGF (20 dips/min); subsequently, the dissolution medium was replaced with FaSSIF for more 180 min (15 dips/min). They demonstrated that both SNEDDSs allowed a faster release rate of hydrochlorothiazide when compared to the free drug. An in vitro release of 27.4% was achieved after 30 min for the hydrochlorothiazide powder, while release rates of 81.9 and 75.6% were achieved by SNEDDS-1 and SNEDDS-2, respectively.

### 5.2. In Vitro Lipolysis

**Figure 6:**
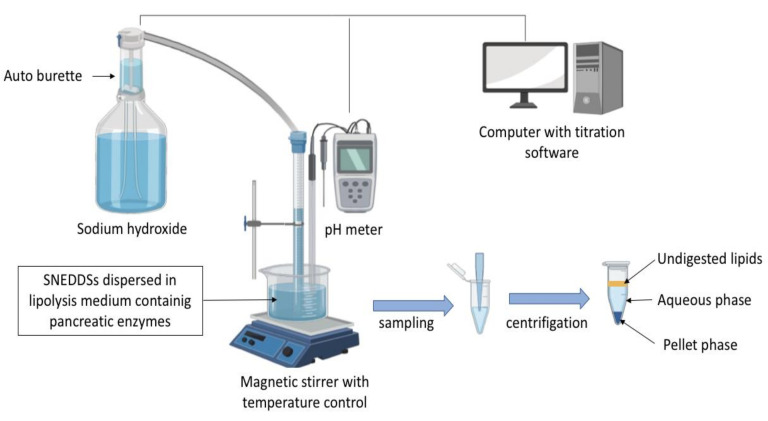
pH-stat lipolysis model for the in vitro assessment of lipid-based drug-delivery systems.

In vitro lipolysis has increasingly been used to assess the likely impact of digestion by gastric/pancreatic enzymes and the dispersion in intestinal fluids of lipid-based formulations, including SEDDSs [73,124,125]. The most frequently employed in vitro lipolysis model to evaluate SNEDDSs is the pH-stat lipolysis model [126,127,128]. The experimental setup generally consists of different equipment used to mimic the intestinal environment, as depicted in Figure 6. The in vitro lipolysis is generally carried out in a thermo-controlled reaction vessel containing a lipolysis medium representative of either fed or fasted GI fluid, formulated with an accurate pH buffer capacity along with bile salt, phospholipids, and NaCl.

The digestion is triggered by addition of pancreatin extract containing lipases and other pancreatic enzymes (amylase, protease, and ribonuclease). These enzymes hydrolyze TG and other digestible SNEDDSs components (i.e., surfactants), which subsequently release free fatty acids. The fatty acids released are automatically titrated with sodium hydroxide to neutralize the drop in pH caused by the enzymatic lipolysis. The addition of calcium is important to form insoluble soaps with free fatty acids and thereby removes them from the system. Free fatty acids could migrate at the oil–water interface and inhibit enzyme activity [129]. Assuming that a high in vitr*o* drug solubilization equals a high oral absorption, the percentage of drug dissolved in the aqueous phase during the in vitro lipolysis has been related to high oral drug absorption [106]. With this relationship, many studies have described rank-order correlation between the patterns of drug solubilization obtained on in vitro lipolysis and the plasma profiles after oral administration [130,131,132,133]. Thus, SNEDDSs that show evidence of drug precipitation during the digestion appear more likely to result in poorer in vivo drug exposure [134]. The additional solid-state characterization of the precipitates (nature/form) formed during SNEDDSs lipolysis may therefore contribute to the improvement of quality of data interpretation. A drug precipitation in amorphous form (or molecular dispersed state) might be expected to lead to rapid in vivo drug re-dissolution in comparation to the precipitation in the crystalline form [135,136,137]. Several techniques can be used to study the solid-state of the precipitates, including UV imaging, X-ray diffraction and in-line Raman spectroscopy [138,139,140,141]. 

Moreover, advances in synchrotron small-angle x-ray scattering (sSAXS) are providing greater details of the real-time structural configuration and colloidal phase transitions of lipolyzed formulations [142,143]. sSAXS has been used to control the structural evolution of colloidal structures on a shorter time scale and drug behaviors (solubilization and/or precipitation) on a longer time scale during lipolysis in real time [144]. This technique avoids the need for sample inhibition, time point collections, extended storage and sample retrieval for test, further improving the accuracy and efficiency of the process [74].

Memvanga et al. [40] developed SEDDSs to increase the oral bio-availability of curcumin. Results from the in vitro lipolysis showed that 90–95% of curcumin remained solubilized (Figure 5b), and X-ray powder diffraction analysis of the pellets revealed that 5–10% of the drug precipitated in amorphous form (Figure 5c). Christophersen et al. [133] evaluated the ability of a GI in vitro digestion model to predict the in vivo performance of two SNEDDSs formulations and a commercial tablet of cinnarizine, both in the fasted and fed states in dogs. A SNEDDS (sesame oil, oleic acid, Brij 97, Cremophor^®^ RH 40, ethanol) was either filled into a gelatin capsule (SNEDDS-A) or loaded onto a porous tablet core (SNEDDS-B) and compared to a commercial tablet in an in vitro digestion model. The results in the fasted state showed that the percentage of dissolved drug decreased in the following order: SNEDDS-A > SNEDDS-B > tablet, which correlated well with the in vivo bio-availability. In the fed state in vitro digestion model, the amount of cinnarizine dissolved was similar for all formulations. The authors noted the increase in conventional tablet performance explained by food effect. The X-ray powder diffraction (XRPD) analysis of the pellets obtained at the end of the in vitro digestion showed that the drug from the commercial tablets precipitated in crystalline forms. Khan et al. [123] coupled in vitro lipolysis with sSAXS to simultaneously monitor the solid-state characteristic of precipitated fenofibrate from the lipolysis of a SNEDDS. Results showed that fenofibrate precipitates in its thermodynamically stable crystalline form upon lipolysis of the SNEDDSs, and an increase in scattering intensity over time corresponded well to an increase in concentration of precipitated fenofibrate in the pellet phase (Figure 5d).

However, while the pH-stat lipolysis model provides one means of predicting the oral absorption, it is a closed system, and many studies have since revealed a lack of in vitro–in vivo correlation (IVIVC) using the same lipolysis model [145,146,147]. Moreover, the lack of the absorption sink that is present in vivo will most likely lead to an overestimation of drug precipitation, which may produce an incorrect estimation of the in vivo performance [148,149].

In an attempt to simulate the in vivo conditions as closely as possible, recent research has developed several digestion models, including a high-throughput lipolysis model [150,151], a Permeapad^®^ lipolysis/permeation model [136], two compartmental simultaneous setups [152,153] and the μFLUX system [107].

### 5.3. In Vitro Permeation Studies

The parallel artificial membrane permeability model (PAMPA) and the Caco-2 cell model are the two most often used to evaluate the drug permeation in vitro [154].

PAMPA is a high-throughput technique, based on an artificial lipidic membrane that is useful in predicting the passive oral drug absorption [155,156]. Initially, drug is placed at the donor compartment, and the apical compartment is drug-free. After the incubation time, the quantity of drug is determined in each compartment. The compartments may also contain some additional ingredients to bind the drug as it permeates [157,158]. PAMPA is especially advantageous in early drug discovery and, since it is easy to automate, cost-effective and compatible for high-amount solubilizers [159,160]. Nekkanti et al. [161] developed SNEDDSs and proliposomes for valsartan and compared their in vitro/vivo performance. SNEDDSs were developed using varying amount of Labrafil^®^ M 2125, Capmul^®^ MCM, and Tween^®^ 80, while proliposomes containing soy phosphatidylcholine, hydrogenated soy phosphatidylcholine, a distearyl phosphatidylcholine were developed by a thin-film hydration technique. Results from in vitro drug permeation studies using PAMPA showed an increase in drug permeability from SNEDDSs and proliposomes over the pure drug. The effective permeability values for the pure drug, proliposomes, and SNEDDSs formulations were found to be 1.0 × 10^−5^, 1.7 × 10^−5^, and 1.8 × 10^−5^, respectively. However, the limitations of PAMPA are that the lipidic membrane is slightly different from the biological membrane and the presence of organic solvent in the membrane, which could result in a non-bilayer membrane structure. Furthermore, PAMPA is limited to passive permeation evaluation [159,162].

The Caco-2 cell line is routinely cultivated as monolayers on permeable filters to study intestinal drug absorption. The drug transport across the GI epithelium cells may occur by several pathways, including the passive paracellular and transcellular routes, the carrier-mediated pathways and transcytosis. Mature Caco-2 cells have been used to study transport of drugs by all these pathways [163,164,165,166]. Although Caco-2 originated from human colon carcinoma, they develop numerous features of absorptive GI cells during culture, such as microvillous structure, hydrolysis enzymes, tight junctions, and carrier-mediated transport system of fatty acids, amino acids, sugars, and many drugs [167,168,169]. Similar to in vivo conditions in intestinal cells, once in contact with lipids, they can synthetize and secrete chylomicrons [170]. Caco-2 cells can be pretreated with different inhibitors to elucidate the uptake mechanisms of drugs and lipid nanocarriers [50,171]. Several studies have shown enhanced drug permeation from SNEDDSs using Caco-2 monolayers [172,173,174,175,176]. Memvanga et al. [40] demonstrated that the transport of the curcumin-SEDDSs across Caco-2 monolayers was improved compared with that of free drug (Figure 5e).

Apart from the permeability assessment, Caco-2 cells could be used to evaluate the safety of many lipid-based formulations. In these assays, Caco-2 cells are treated with increasing amounts of the formulation dispersed in a suitable buffer and left to incubate. Many cellular processes such as DNA synthesis metabolic activity and proliferation can be used to evaluate cell viability after the incubation [177,178,179]. Widely used in vitro cytotoxicity assays include 3-(4,5-dimethylthiazol-2-yl)-2,5-diphenyltetrazolium bromide (MTT), 3′-[1-[(phenylamino)-carbonyl]-3,4-tetrazolium]-bis(4-methoxy-6-nitro)benzene-sulfonic acid hydrate (XTT) and 3-(4,5-dimethylthiazol-2-yl)-5-(3-carboxymethoxyphenyl)-2-(4-sulfophenyl)-2*H*-tetrazolium (MTS), which give direct indications of cell viability and proliferation [180,181,182,183]. These assays are based on mitochondrial reduction of tetrazolium salts to dyed formazan-based products, providing information on cell activity and metabolism [184]. The main differences between them rely on the chemical compositions of tetrazolium salts. MTT is a positively charged compounds that easily diffuses viable cells and converts to insoluble formazan products, whereas MTS and XTT are negatively charged compounds that are readily transformed into soluble formazan products [154]. 

Lactate dehydrogenase (LDH) assay is commonly used to access cell membrane damage [177]. The activity of LDH released in the cell culture medium after nanoparticle treatment is spectrophotometrically measured. Released LDH converts pyruvate into lactate resulting in the chemical reduction of NADH into NAD^+^. A drop in NADH absorption peak correlates to an increase in extracellular concentration of LDH [154].

Desai et al. [185] compared the cytotoxicity of MCT- and LCT-containing SEDDSs on Caco-2 cells of varying maturity (1-, 5-, and 21-day cultures). The cell viability was determined using MTT assy. They demonstrated that the oil-surfactant mixtures had greater tolerance than surfactants alone, and LCT-SEDDSs were well-tolerated at almost 10-fold higher concentrations than the corresponding MCT-SEDDSs. Moreover, the LCT-SEDDSs showed better tolerance compared to MCT-SEDDSs after lipolysis. The authors concluded that MCT and LCT lipids are well-tolerated at normal human dose, and LCT lipids were less toxic than MCT lipids in a Caco-2 cell model.

## 6. Ex Vivo Permeation Studies

Intestinal absorption has been recognized as a crucial factor affecting the plasma concentration of compounds loaded in SNEDDSs. Several isolated systems have been used to determine the GI absorptive ability of a drug and the mechanism behind this process. These systems contribute to the reduction of live animal in experimentation. Frequently used systems are single-pass intestinal perfusion (SPIP) and intestinal recirculating perfusion that provide conditions closer to what is faced after oral ingestion [185,186,187]. The SPIP is based on the principle that the amount drug in perfusion nano-emulsion decreases over time due to the drug permeation [188]. It allows the determination of the rate and extent of permeation through the intestinal segment (i.e., duodenum, jejunum) after cannulating at both ends. The SPIP is advantageous for compounds that are rapidly absorbed [46,189]. In the intestinal recirculating perfusion (IRP), the process is repeated many times with the same perfusate. Due to the longer retention time within the intestine, the probability of drug absorption is considerably increased. Then, it is dedicated to drugs that are absorbed slowly to amplify the concentration change [190,191].

Kazi et al. [192] investigated the in vitro and in vivo performance of SNEDDSs loaded with talinolol. The in vitro dissolution revealed a significantly higher drug dissolution rate from SNEDDSs (>92% in 2 h) compared to pure drug. The data from in vitro lipolysis showed that SNEDDSs presented comparably higher amounts of drug in aqueous phase under both fed and fasted (60% and 67%, respectively) conditions. The ex vivo permeability by SPIP showed a 4-fold increase in permeability from SNEDDSs compared to pure drug. In another study, Beg et al. [193] used the quality-by-design (QbD) approach to design and optimize SNEDDSs of paclitaxel with improved biopharmaceutical attributes. Following appropriate mathematical models, the optimized SNEDDSs were earmarked by QbD optimization. Next, cationic SNEDDSs were formulated for both LCT- and MCT-containing SNEDDSs and were subjected to in vitro testing. The in vitro dissolution study indicated a 2.7-fold enhancement in dissolution rate from optimum cationic SNEDDSs over free drug. Ex vivo SPIP study exhibited nearly 6- to 8-fold enhancement in absorption and absorption parameters of the drug from the optimized cationic SNEDDSs as compared to the pure drug.

## 7. In Vivo Pharmacokinetics Studies

In addition to primary in vitro studies, animal pharmacokinetics studies play a major role in predicting the oral bio-availability in humans during drug development [194]. Generally, an oral dose of the drug loaded in SNEDDSs are given to animals (preconcentrate or dispersed in water). To analyze the absorbed drug in the plasma, various analytical techniques such as liquid chromatography-UV and liquid chromatography-mass spectrometry are commonly used [137,195]. The pharmacokinetic parameters (i.e., t^1/2^, C_max,_ T_max,_ AUC_0-t_) from animals are extrapolated to humans to select a suitable dose to use during the first trials in humans. When compared to dogs, rabbits, or pigs, rats are an economical, convenient, and relatively high-throughput animal model. Another advantage of rats is the potential of inhibition of efflux pumps, transporters, and enzymes, allowing the evaluation of their impact on drug absorption [85]. However, it should be noted that one of the major issues of extrapolating bio-availability from animals to humans is the fact that the anatomy and physiology of animals vary largely; therefore, the oral absorption of a drug dose varies across species.

There are hundreds of published articles on pharmacokinetics studies with SNEDDSs in animals such as rats, dogs, or rabbits. Diverse SNEDDSs have been formulated and have shown superior in vitro/in vivo performance compared with native drugs. Some preclinical studies reporting enhanced bio-availability from SNEDDSs formulations are presented in Table 3, with a brief description that gives an overview of this field.

Aside from improved oral absorption, SNEDDSs have been reported to minimize the impact of food effect and bile secretion on the oral drug absorption [84,113]. Perlman et al. developed SEDDSs that provided considerably higher fasted exposures of torcetrapib than the formulation containing Miglyol^®^ 812, previously employed in the clinic. SEDDSs composed of 30% Capmul^®^ MCM, 20% MCT, 30% Triacetin, and 20% Polysorbate 80 enhanced fasted exposure and thus decreased the effect of food from 5- to 3-fold in dogs at a dose of 90 mg [221]. Moreover, reduced intra- and inter-subject variabilities by SNEDDSs were reported [84,222].

In contrast, a literature review revealed fewer clinical studies in which the absorptions of drugs were enhanced by administration in the form of SNEDDSs. Some examples are given here, and Table 4 summarizes them. 

**Figure 7:**
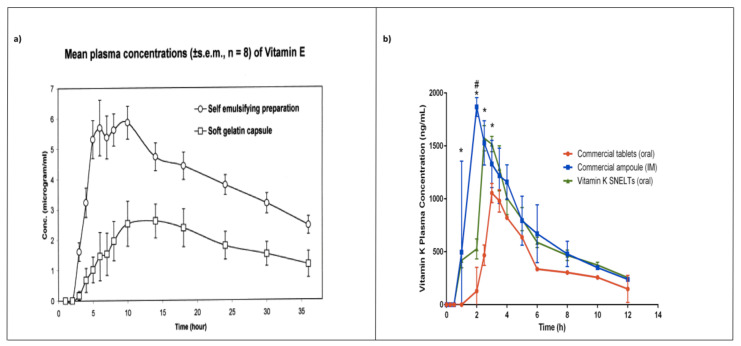
(**a**) Mean plasma concentration (±SEM, *n* = 8) of a-tocopherol as a function of time following oral administration of vitamin E (400 IU) in the form of a self-emulsifying preparation and soft gelatin capsule after subtraction of endogenous vitamin E from each subject. From [223]. (**b**) Plasma concentration-time profiles of vitamin K after intramuscular and oral administration of commercial vitamin K products and oral administration of vitamin K SNELTs to human volunteers. Each value represents the mean ± SD (*n* = 6). * *p* < 0.05 compared to the commercial vitamin K tablet (oral); # *p* < 0.05 compared to the commercial vitamin K ampoule (IM). From [222].

Julianto and colleagues [223] conducted a single-dose study to evaluate the oral bio-availability of a ∝-tocopherol SEDDS in comparison with that of a commercial product, Natopherol^®^, available as soft gelatin capsules. The SEDDS contained 40% palm oil, 20% Span^®^ 80, 40% Tween^®^ 80, and alpha-tocopherol (333.3 IU/mL), whereas the commercial formulation contained alpha-tocopherol (400 IU) dissolved in soybean oil. They demonstrated that SEDDS formulation enhanced the oral bio-availability of alpha -tocopherol between 210 and 410% compared with the commercial formulation in healthy male volunteers (Figure 7a).

Postolache et al. [224] compared the oral bio-availability of cyclosporine SEDDSs with a marketed semi-solid oily solution cyclosporine on 24 human healthy volunteers. The results showed that both the AUC_0-∞_ and C_max_ values of the SEDDSs were higher than those of the oily solution. The authors concluded that the oily solution was not bioequivalent with the SEDDSs formulations owing to the lower absorption rate.

A comparative pharmacokinetic study was conducted to evaluate the oral bio-availability of tocotrienols from SEDDSs and an oily solution. Liquid formulations loaded with 200 mg mixed tocotrienols administrated in healthy adults as SEDDSs or simple solution of soybean oil stated that SEDDSs showed a rapid onset of absorption, with a marked increase in the extent of the drug bio-availability by almost three-fold compared to the soybean oily solution under fasted condition [82].

Roche Laboratories enrolled human subjects to compare the bio-availability of Fortovase^®^ and Invirase^®^, both available in the market as soft and hard gelatin capsules, respectively. Fortovase^®^ was a SEDDS containing saquinavir (200 mg) dissolved in medium-chain mono and di-glycerides, povidone and ∝-tocopherol, whereas Invirase^®^ contained saquinavir (500 mg), microcrystalline lactose, sodium starch glycolate, povidone, magnesium stearate, and talc. The study demonstrated a significant improvement of the oral bio-availability up to 331% from Fortovase^®^ compared with Invirase^®^ [226]. Due to pill burden and GI tolerability issues, Fortovase^®^ was later discontinued from the market [226].

The pharmacokinetic parameters of vitamin K self-nano-emulsifying lyophilized tablets (SNELTs) were evaluated and compared with marketed tablets and ampoules on human volunteers [222]. SNELTs enhanced vitamin K’s relative bio-availability (170%) in comparison with the marketed tablets. Moreover, promisingly, SNELTs showed no statistically significant difference in the AUC compared with the marketed IM injectable ampoules (Figure 7b).

## 8. Advancements in SNEDDSs

### 8.1. Supersaturated SNEDDSs

Drug solubility in lipidic components is the key factor that determines the dose of a drug to be administered in a SNEDDS formulation [227,228]. As the oil content is reduced during the dispersion or digestion, the solubilizing capacity of SNEDDSs declines in vivo*,* leading to drug precipitation [229]. Therefore, most SNEDDSs contain drugs below their equilibrium solubility, typically between 50% and 90%, limiting the access of many drugs to this promising technology, especially drugs that should be given at a high dose [230,231,232].

To overcome this drawback, supersaturated SNEDDSs (s-SNEDDSs) containing precipitation inhibitors have been suggested [228]. s-SNEDDSs are thermodynamically stable SNEDDSs containing a polymer (such as poly vinyl pyrrolidone (PVP) or hydroxypropylmethylcellulose) that should inhibit the nucleation process and subsequent drug precipitation, thus temporarily maintaining a supersaturated solution of the drug in the GI tract [233,234,235]. Supersaturation enhances the thermodynamic stability of the drug above its solubility limit, thus improving both the extent and rate of drug absorption [3]. Moreover, the higher drug loading in the formulation increases the flux over the GI epithelium [236]. Bannow et al. studied the impact of the polymeric precipitation inhibitor (polyvinylpyrrolidone-co-vinyl acetate) PVP/VA-64 on the in vitro performance and physical stability of s-SNEDDS containing simvastatin. They demonstrated that s-SNEDDSs containing 20% (*w*/*w*) of PVP/VA-64 and a simvastatin load of 200% enhanced formulation performance during in vitro digestion, achieving a 2.5-fold higher degree of drug supersaturation after 15 min of lipolysis in comparison with PVP/VA-64-free s-SNEDDSs of the same simvastatin load [237].

As per the literature, many researchers have indicated that the bio-availability of a drug in s-SNEDDSs is enhanced and is greater than that in the traditional SNEDDSs [232,238]. s-SNEDDSs have also been employed to reduce the oil/surfactant content in the conventional SNEDDSs formulations. The high concentrations of these surfactants typically present in SNEDDSs can lead to GI side effects. It has been noted that the significantly reduced amount of oil/surfactant in s-SNEDDSs offers an improved safety/toxicity profile than the classical SNEDDSs [231].

### 8.2. Mucus-Permeating SNEDDSs

Due to faster clearance rates and rapid secretion, the mucus barrier sets a challenge for conventional drug-delivery systems to reach the GI epithelial cell surface and remain there for a sufficient amount of time [239,240]. It has been reported that SNEDDSs composition and resulting nano-emulsion droplet size are the most important factors influencing the mucus-permeating ability of a SNEDDS formulation [241,242]. Most SEDDSs formulations contain surfactants made of PEGylated groups to ensure self-emulsification process, so their relatively high mucus-permeating abilities can be explained by those PEGylated groups located at the surface of the oil droplets, making SNEDDSs highly muco-inert [243].

Friedl et al. observed the permeation of different droplet-sized SNEDDSs across mucus membranes and demonstrated that SNEDDS with a particle size of 12 nm had greater diffusion potential (70%) compared to the diffusion (8%) of the large SNEDDS (450 nm) [244].

Currently, several strategies are used to improve mucus permeation of SNEDDSs, including surface charge modification [245,246,247,248], mucoadhesive polymer incorporation [249,250] and the inclusion of mucolytic agents [251,252].

SNEDDSs that can change their zeta potential from negative to positive were formulated. Those SNEDDSs containing highly phosphorylated molecules have a negative zeta potential and change their zeta potential to positive once coming into contact with intestinal alkaline phosphatase, an enzyme presents in the GI mucus gel layer [91,246]. The advantages of this approach are that negatively charged SNEDDSs formulation can diffuse more quickly across the mucus gel layer, and zeta potential are shifted to positive once in contact with GI epithelium, allowing improved cellular uptake.

Mucoadhesive SNEDDSs are developed to prolong nanoparticle residence time at GI epithelium surfaces, thus avoiding pre-systemic drug metabolism. The choice of an appropriate mucoadhesive polymer in terms of lipophilic properties and compatibility is primarily important. The classical polymers (e.g., carboxymethyl cellulose, chitosan) adhere by forming hydrogen bonds or weak electrostatic interactions, resulting in a relatively low muco-adhesion, generally insufficient to ensure a prolonged localization at a specific target site [13]. To address this issue, thiolated polymers were introduced as a new generation of mucoadhesive polymers [249,250]. In contrast to classical mucoadhesive polymers, these novel polymers have the capability of enhanced attachment via covalent bonding [253]. Leonaviciute et al. provided a proof-of-concept that mucoadhesive Self-Emulsifying Drug Delivery Systems (SEDDS) can be obtained using hydrophobic mucoadhesive polymers. A thiolated Eudragit^®^ S100 was synthesized and incorporated into SEDDSs (T-SEDDSs). They demonstrated that T-SEDDSs led to markedly improved muco-adhesiveness compared with blank SEDDSs. Blank SEDDSs were totally removed from the GI mucosa after 15 min, whereas more than 60% of T-SEDDSs were still attached to it [254].

Mucolytic agents can improve the SNEDDSs permeation across the GI barrier by breaking down certain three-dimensional substructures within the mucus network [88,255]. Instead of cleaving the entire mucus network and consequently its important protective role, these agents break down the mucus gel layer only where they are in contact with it. Leichner et al. developed SEDDS with mucolytic properties following the incorporation of papain. The enzyme was encapsulated into SEDDS via hydrophobic ion pairing technique using sodium deoxycholate. The formulated SNEDDS exhibited an almost 3-fold increase in mucus diffusion and an extended residence time at the mucosal (up to 3- and 5-fold) compared to the control [256].

### 8.3. Solid SNEDDSs

Despite the benefits provided by liquid SNEDDSs, drawbacks such as drug/components precipitation when stored, interactions between the filling and the capsule shell, and formulation stability during storage are common issues faced by them [27,28,257]. The main strategy applied to overcome these challenges is to transform liquid SNEDDSs into solid dosage SNEDDSs formulations. It is believed that the conversion of liquid SNEDDSs to solid SNEDDSs provides relatively lower production cost, better formulation stability, ease of handing, precise dosing, and, consequently, better patient compliance [258,259,260,261]. 

Generally, the techniques employed to develop solid SNEDDSs include adsorption onto inert carriers [262,263], spray drying [264,265], melt granulation [266,267] and extrusion-spheronization [268] and are described below.

#### 8.3.1. Methods of Production

Adsorption onto solid carriers with high specific area and/or high porosity is the most studied technique to produce solid SNEDDSs [269]. The process of adsorption is easy and just implies addition of the liquid SNEDDSs onto solid porous carriers with gentle mixing in a blinder. The solid porous carriers commonly used include silicates such silicon dioxide (i.e., Aerosil^®^), sodium carboxymethylcellulose, micronized porous silica (i.e., Syloid^®^), hydroxylpropyl beta cyclodextrin, and magnesium stearate. The obtained solid SNEDDS can directly be filled into gelatin capsules or, alternatively, mixed with appropriate ingredients prior compression into tablets. Benefits of this technique include the avoidance of organic solvents and the small number of excipients required for the formulation [270]. Furthermore, liquid SNEDDSs can be adsorbed at high levels (>60% *w*/*w*) onto a suitable solid carrier [271]. 

Spray drying is a simple one-step technique for producing solid micro/nanoparticles including solid SNEDDSs [272]. The solid carrier is mixed with the liquid component using a solvent followed by solubilization. The solubilized liquid formulation is then sprayed into a hot-air compartment to remove the volatile solvents, which can be organic solvents or water in the case of nano-emulsion. Dried particles under controlled temperature and flow rate are prepared. Such micro/nanoparticles can be further filled into capsules or converted into tablets.

Solid SNEDDSs production by spray-drying technique is feasible with several solid carriers, whether hydrophobic or hydrophilic. The choice of a solid carrier can impact the release profile and the oral absorption of the drug by affecting the formulation droplet size and entrapment after reconstitution [273,274,275]. The sprayer, the airflow, the chamber temperature, and the design of drying chamber are chosen with the respect to the powder specifications and product drying characteristics.

Melt granulation is a technique in which agglomeration of powder is obtained through the addition of a softening or binder at low temperature (50–80 °C). The melted binder establishes liquid bridges between particles and forms small granules that are transformed into spheronized pellets under specific conditions. Generally, 15–25% of the binder can be used depending on the powder fineness [276]. Melt granulation offers several advantages in comparison to conventional wet granulation, as it is a simple operation, in which the addition of the liquid component and the subsequent drying phases are excluded. The process parameters to be considered include mixing time, binder particle size, viscosity of the binder on melting and impeller speed [277,278].

Extrusion/spheronization is one of the pelletization methods employed in the pharmaceutical industry to manufacture a series of solid dosage forms, including pellets, granules and tablets [272,279]. Extrusion is technique used to convert a raw material with plastic characteristics into a product of uniform shape and density by forcing it through a die under controlled conditions of temperature, pressure, and product flow. Extrusion is then followed by a spheronization process, in which the product (extrudate) is broken and transformed into round pellets [280]. The following steps are applied during extrusion/spheronization process: mixing of the liquid SNEDDSs and components including adsorbent to form a monogenous powder; wet massing binder; extrusion into a spaghetti-like product; spheronization from the extrudate to spheroids of uniform particle size; drying; and sifting to achieve the desired particle size distribution. The characteristics of the SNEDDSs pellets formed greatly depend on the pellet composition. A balance between the smallest quantity of absorbent required and the largest quantity of liquid SNEDDSs is necessary to formulate pellets with desired biopharmaceutical attributes and the highest possible drug loading [281,282]. The drawbacks of this technique often include the high-energy input manly attributed to temperature and shear forces.

#### 8.3.2. Solid-State Characterization of Solid SNEDDSs

In addition to characterization techniques used for liquid SNEDDSs, solid SNEDDSs require further specified solid-state characterization. The inner physical structure of the powder particles is mainly verified by thermal analysis and X-ray diffraction, while different component interactions are studied by Fourier-transform infrared spectroscopy (FTIR).

Differential scanning calorimetry (DSC) and thermal gravimetric analysis (TGA) are mostly used to evaluate the thermal behavior of solid SNEDDSs. In these techniques, samples are subjected to heating at a specified temperature rate under different atmospheres such as argon, oxygen, and nitrogen [54]. The main information generated from these techniques is the melting point, crystallinity, polymorphism, and endothermic and exothermic behaviors of the sample, which are related to a reference standard [283]. 

X-ray diffraction is used to determine the crystallization and polymorphism of drugs in solid SNEDDSs [284]. Most drugs, lipids and surfactants have several polymorphic forms that can change after the encapsulation [285]. Since the biological activity of molecules also relies on polymorphic form, it is very important to ensure the stability of an appropriate form after the solidification. The X-ray pattern of the encapsulated drug is compared with the reference, and any difference indicates the impact of the solidification process on the drug stability.

FTIR is used to analyze intermolecular interactions and drug-carrier compatibilities. It provides information about functional groups and different chemical bonding between molecules. FTIR allows a study of the functional and structural modifications during the formulation and possible interactions between oils, surfactants, cosurfactants/cosolvents and molecules [54,228].

### 8.4. SNEDDSs for the Oral Delivery of Hydrophilic Macromolecules

The use of hydrophilic macromolecules (polysaccharides, peptides, protein and genes) has attracted growing interest presently owing to their high specificity, selectivity, and reduced side effects. Currently, more than 120 biopharmaceuticals, especially proteins, are approved for the use in clinic by the US Food and Drug Administration [286,287,288]. However, there are many challenges towards the oral administration of these hydrophilic macromolecules due to GI barriers that limits their oral absorption [289]. The low oral bio-availability of these drugs is a result of many factors, including poor diffusion related to hydrophilicity and large size, mucus barrier, gastric acidity, and enzymatic degradation [241,290].

Advanced SNEDDSs provide novel nano-emulsions with improved functional characteristics such as prolonged GI residence time, increased stability in GI fluids, improved mucus diffusion, improved permeation, and enhanced cell uptake, leading consequently to increased oral bio-availability of the encapsulated drugs [10,11,291,292]. Bravo-Alfaro et al. [293] developed SNEDDSs containing an insulin complex along with modified or unmodified phosphatidylcholine to increase insulin oral bio-availability. Under *i*n vitro GI conditions, SNEDDSs showed 35.7% of drug availability upon reaching the final stage of the simulated small intestine. In vivo studies using diabetic rats showed a 36.1% decrease in plasma glucose levels after 4 h of SNEDDS administration and only 1.8% bio-availability after subcutaneous insulin administration. 

SNEDDSs are also considered to be an innovative alternative for oral delivery of gene among the non-viral vectors. Incorporation of nucleic acids (e.g., pDNA, siRNA, microRNA) into nano-emulsions formed upon SNEDDSs dispersion could protect them from enzymatic metabolism and enhance their cellular uptake [294,295]. Mahmood et al. [291] loaded a pDNA into SNEDDS formulation as a pDNA/cetrimide complex at a molecular ratio of 1/2. Furthermore, the transfection efficiency was improved by encapsulating HIV-1 Tat protein (a cell-penetrating protein). The transfection efficiency tested on HEK-293-cells was found to be 1.7 and 1.8-fold higher for SNEDDSs loaded with Tat protein in comparison to Lipofectin and control, respectively.

However, the incorporation of any hydrophilic drug in the oily phase of a SNEDDS is difficult due to its low lipid solubility, which is responsible for low drug loading [246,296]. Several strategies have been developed to increase the lipid solubility of hydrophilic macromolecular drugs to facilitate their encapsulation into SNEDDSs formulations. These strategies are presented here:

#### 8.4.1. Ion Pairing

The hydrophobic ion paring process for hydrophilic drugs is based on partial or full binding of the drug with an amphiphilic ligand with opposite charge, or lipophilic pro-drug to increase the hydrophobicity and lipid solubility [297]. This association is a reversible and cost-effective method that enhances the lipid solubility along with the ability to cross membranes without changing the native structure of the molecule [297]. The potential of this method in the oral delivery of hydrophilic drugs has been examined in vitro and in vivo. Menzel et al. demonstrated the high impact of ion paring on the oral bio-availability of exenatide. Exenatide was lipidized via hydrophobic ion pairing with sodium docusate (DOC) and encapsulated into a SEDDS consisting of 25% Labrafil^®^ 1944, 30% Capmul^®^-PG 8, 35% Cremophor^®^ EL, and 10% propylene glycol. The results from in vivo evaluation in rats showed a 14.6-fold higher relative bio-availability versus subcutaneous exenatide solution [210]. In another study, Hauptstein et al. [290] successfully encapsulated pDNA as a hydrophobic ion-paired complex with different cationic lipids into a SNEDDS formulation composed of 30% Capmul^®^ MCM, 30% Captex^®^ 355, 30% Cremophor^®^ EL, and 10% propylene glycol. In vitro degradation studies via DNase I revealed that pDNA encapsulation into SNEDDS formulation led to significantly prolonged resistance time against enzymatic degradation (up to 8-fold) in comparison to pDNA–lipid complexes and naked pDNA. Furthermore, transfection studies showed a significantly improved transfection efficiency compared to naked pDNA. Many other amphiphilic molecules have been studied for hydrophobic ion paring and encapsulation into SNEDDSs [63,298,299,300,301]. Indeed, soybean phosphatidylcholine remains the most largely investigated amphiphilic molecule to facilitate the encapsulation of hydrophilic drugs into SNEDDSs. 

#### 8.4.2. Double Emulsification Technique

**Figure 8:**
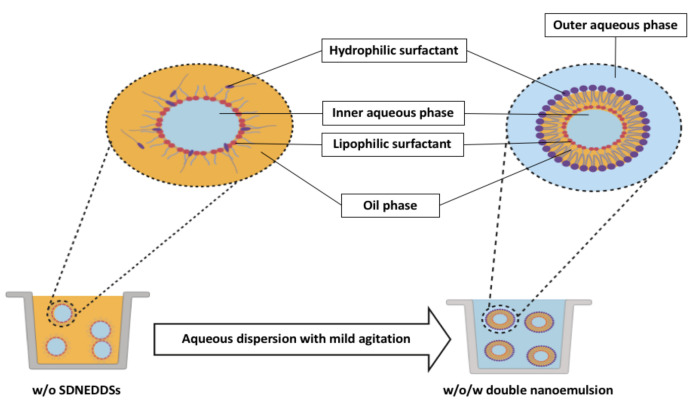
Schematic illustration of the double emulsification technique. Adapted from [205].

Double emulsification technique is an alternative method used to encapsulate hydrophilic drugs into SNEDDSs via the formation of self-double nano-emulsifying drug-delivery systems (SDNEDDSs), as depicted in Figure 8.

In this process, hydrophilic drugs are first dissolved in the inner water phase, whereas lipophilic excipients are dissolved in lipids. The water phase is then dispersed in the oily phase to form preconcentrate w/o SDNEDDSs. SDNEDDSs undergo self-emulsification to w/o/w double nano-emulsion upon aqueous dispersion in the GI tract [302]. SDNEDDSs can save protein and other macromolecular drugs from enzymatic degradation in the GI tract, improve efficacy and reduce the drug dose [303,304,305]. However, the drug stability in the inner water phase always represent a challenge.

#### 8.4.3. The Use of Hydrophilic Cosolvent

The use of a suitable cosolvent can facilitate the incorporation of hydrophilic macromolecular drugs into SNEDDSs formulations. The popular cosolvents used include propylene glycol, polyethylene glycol (PEG)-400, glycerol, and ethanol as mentioned earlier. They enhance the solvent capacity of the formulation and increase the dispersibility of surfactant in the oily phase, thus promoting SNEDDSs homogeneity and stability. Winarti et al. [306] used glycerin as a cosolvent to incorporate bovine serum albumin into a SNEDDS formulation. Bovine serum albumin was first dissolved in glycerin and then encapsulated into the oil phase using surfactants that have HLB values ranging between 11–15. However, there are several limitations related to these cosolvents, including the incompatibility of low-molecular-weight cosolvents with capsule shells and immiscibility of some of them with oils [307].

#### 8.4.4. Chemical Modification

One attractive strategy for improving the solubility and diffusion properties of hydrophilic macromolecular drugs is to combine them with membrane-binding carrier molecules. Hydrophobic carrier molecules such as fatty acids are among the most potentially useful categories of carriers, and studies showed that fatty acid-conjugated peptides and proteins may cross cell membranes, including the blood-brain barrier [308,309].

To develop a Bowman–Birk protease inhibitor (BBPL) into an effective cancer chemo-preventive agent, Wang et al. developed a technique to prepare a reversibly conjugated BBPL with palmitic acid (PA-BBPL) [310]. The results of the study showed that pharmacokinetic parameters of PA-BBPL were largely different from those of free BBPL. An extended plasma half-life and increase (11-fold) in the AUC_0-∞_ were observed for the lipidized form of BBPL. In addition, owing to the reversibility of the combination, PA-BBPL was showed to be equally potent as the free BBPL in the prevention of carcinogen-induced transformation of C3H10T1/2 cells in culture [311]. In another example, the lipophilicity of dalargin was enhanced by 0-esterification of tyrosine with palmitic acid. Dalargin-palmitic acid complex (DL-PA) was encapsulated into selected SEDDSs composed of 40% Cremophor^®^ EL, 50% Capmul^®^ 90, and 10% propylene glycol and SEDDS composed of 30% Capmul^®^ MCM, 30% Captex^®^ 8000, 30% Cremophor^®^ EL, and 10% propylene glycol. Both SEDDSs showed significant mucus-permeating potential as well as protective effects against dalargin degradation by trypsin, elastase and α-chymotrypsin [312].

### 8.5. Targeted SNEDDSs

Drugs in clinical trials may fail to reach favorable outcomes because they cannot target a desired site of action. A successful strategy to overcome this issue is to develop targeted drug-delivery carriers that release the drugs at a specific site of action [313]. SNEDDSs can be considered for this approach. Surface-modified nano-emulsions have been developed to reach animals and human liver in a similar way to chylomicrons [314,315]. SNEDDSs can drastically increase the concentration of the drug in liver and/or spleen and can be a smart way to reach these organs [316,317,318].

Another key aspect of SNEDDSs is their ability to be taken up into the lymphatic system. Many diseases, including HIV, lymphoma, autoimmune diseases, leukemia, tissue rejection, and tumor metastasis, require the lymphatic system for their progress [4,319,320,321]. Furthermore, passive and active targeting is achievable by attaching suitable ligands (antibodies, nucleic acid or peptides) to target a specific site of action [322,323]. Batool et al. developed a papain-grafted S-protected hyaluronic acid-lithocholic acid co-block (P-G-S-P-H-L-AC) amphiphilic polymer as a muco-permeating stabilizer to target MCF-7 breast cancer epithelial cells. The P-G-S-P-H-L-AC amphiphilic polymer was incorporated into a SNEDDS loaded with tamoxifen. An ex vivo permeation study revealed 7.11-fold higher diffusion of tamoxifen by tamoxifen P-G-S-P-H-L-AC SNEDDS compared to free tamoxifen. Furthermore, the formulated SNEDDS was safe and compatible against macrophages. It could efficiently kill MCF-7 breast cancer cells compared to free drug [45].

### 8.6. SNEDDSs for the Oral Delivery of Herbal Drugs

Presently, the use of herbal medicines has been increased owing to their therapeutic effects and fewer side effects compared with synthetic drugs [46,324,325,326]. It has been estimated by the World Health Organization (WHO) that more than 70% of the world′s population, mostly in low-income countries, rely on plant medicines for primary health care [327,328,329,330]. Worldwide, plant medicines represent approximately 25% of the pharmaceutical arsenal [331]. However, most of herbal extracts and herbal drugs exhibit poor in vivo activity related to their low solubility, poor gastric stability, high metabolism and, hence, poor bio-availability [22,332,333]. Thus, SNEDDSs represent a very attractive drug-delivery carrier for herbal medicines.

Qian et al. developed SNEDDSs of myricetin to improve its solubility and oral absorption. These myricetin-SNEDDSs had high solubility, fast drug release characteristics (>80% in 1 min), improved permeability and low cytotoxicity compared with the free myricetin. The oral bio-availability of myricetin was improved 2.5- to 6.3-fold compared to myricetin alone in rats [22]. To improve the aqueous solubility and oral absorption of bruceine D, Dou et al. [334] developed a SNEDDS composed of Solutol^®^ HS-15, MCT, and propylene glycol. Bruceine-D-SNEDDS exhibited improved pharmacokinetic parameters as compared with the suspension. Furthermore, bruceine D-SNEDDS formulation significantly restored the body weight and colon length, reduced the disease activity index and colon pathology in a rat model. Tung et al. [335] developed and optimized s-SNEDDSs for the oral delivery of silymarin. s-SNEDDSs containing silymarin, Labrafil^®^ M 1944, Kolliphor RH40 and Transcutol^®^ HP were prepared, and Poloxamer 407 was chosen as the optimal precipitation inhibitor. The relative bio-availability of s-SNEDDSs versus Legalon^®^ (silybum marianum) determined in mice was approximately 760%. Furthermore, s-SNEDDSs revealed a significantly higher hepatoprotective activity in CCl_4_-induced model in contrast to the commercial product and decreased the plasma levels of lipid peroxidation and transaminases along with glutathione and superoxide dismutase activities under tested doses calculated as silybin. Shanmugam et al. used a spray-drying technique to prepare solid SNEDDSs for the oral delivery of the bioactive carotenoid lutein. The solid SNEDDSs contained 25% phosphatidylcholine, 60% Labrasol^®^, 14% Transcutol^®^ HP, and Aerosil^®^ 200 as the inert solid carrier. The pharmacokinetic evaluations performed in rabbits resulted in increased values of *C*_max_ and AUC_0-t_ of carotenoid lutein loaded in solid SNEDDSs. The enhancements of *C*_max_ for solid SNEDDSs were approximately 21- and 8-fold compared with free lutein (FL) and marketed product (MP), respectively. The relative bio-availability of solid SNEDDSs compared with MP and FL were 2.7- and 11.8-fold, respectively [336]. Recently, Kazi et al. designed solid SNEDDSs consisting of curcumin and piperine by incorporating bioactive natural oils (avocado, apricot, black seed and *Zanthoxylum rhetsa*) in the formulations. The optimal liquid SNEDDSs were solidified using Aeroperl^®^ or Neusilin^®^. SNEDDS consisting of 20% black seed oil, 20% Imwitor^®^ 988, 10% Transcutol^®^ HP, 50% Cremophor^®^ RH40 and Neusilin^®^ enhanced curcumin and piperine release (up to 60% and 77%, respectively). In addition, these formulations could efficiently deliver the black seed oil to the patient [337]. Many other studies showed the potential of SEDDSs in the oral delivery of herbal drugs, including [338,339,340,341,342,343,344].

## 9. Challenges

Even though SNEDDSs show considerable benefits over available drug-delivery systems today, still there are aspects that need to be further studied to make SNEDDSs future drug carriers. 

Certain biopharmaceutical issues involving SNEDDSs include the drug-loading capacity and risk of precipitation upon dispersion or digestion. As mentioned above, the formulation-loading capacity could be improved via s-SNEDDSs. The risk of drug precipitation upon aqueous dilution could be minimized by keeping a good balance between oil and the surfactant/cosolvent ratio during the formulation. Many studies demonstrated that small changes in SNEDDSs composition are not expected to bring huge changes in drug solubility, but there could be a crucial decrease in formulation solvent capacity following aqueous dispersion [345,346]. For many years, SNEDDSs that showed evidence of drug precipitation upon aqueous dispersion or digestion appeared more likely to result in lower in vivo drug absorption. This thought process led to widespread use of in vitro dissolution and lipolysis test to evaluate performance of SNEDDSs using GI simulated fluids and the overarching assumption that a high water solubilization in vitro equals a high oral absorption. Although this assumption remains true for several drugs, for certain drugs (i.e., fenofibrate), oral absorption may still be consistent, even in light of notable drug precipitation. Accordingly, supersaturation rather than solubilization is emerging as an important drug driver flux across absorptive membranes [85,128,235,347].

The use of lipids and surfactants as excipients of SNEDDSs requires special attention regarding their safety after oral administration. First, the amount of these excipients in a SNEDDS is usually very high, and second, due to the complexity of their characteristics, these components can create multiple interactions and reactions with the physiological environment that could be difficult to control in vivo. More mechanistic studies will need to be performed to track these ingredients and the potential interactions involved after their ingestion. Moreover, such components, especially surfactants, should be identified as safe, less toxic, and compatible, even at high amounts [348,349]. It is also worth to highlight some drawbacks to which much major attention should be paid, such as the interaction drug-excipients and phenomenon associated with lipid oxidation [350,351,352]. Currently, drug-delivery research groups are working to surmount the aforementioned issues.

In the field of solid SNEDDSs, absorbency, an indicator of the ability to carry greater amounts of liquid SNEDDSs should be searched. The advantages attributed to converting liquid SNEDDSs into solid dosage forms should be weighed against any potential decline in biopharmaceutical performance brought by the solidification process. The development of such SNEDDSs requires better understanding of SNEDDS factors (oil, surfactant, cosolvent, absorbent, etc.) that might impact the biopharmaceutical performance of the products. Accordingly, the implementation of a QbD approach is useful in the development of SNEDDSs as it takes several parameters.

Although the harmonization and standardization of several efficient in vitro tests such as lipolysis have already been established, the knowledge about the in vivo pharmacokinetic parameters and processes involved after SNEDDSs administration remains a gray area, especially in human volunteers [124]. Understanding the in vivo pharmacokinetic parameter is helpful in designing both optimized SNEDDSs and in vitro robust models that can be employed to predict in vivo characteristic accurately, thereby establishing the IVIVC [24,353]. To date, modeling of IVIVC is being increasingly applied as a prediction tool of drug plasma concentration versus time from the in vitro data [131,354,355]. Both processes of formulation dispersion and digestion from SNEDDSs have been grouped into a mathematical model using a series of differential equations [356,357].

Out of all the in vitro tests commonly available for SNEDDSs evaluation, in vitro lipolysis test is found to be more relevant for predicting the in vivo behavior, even though it still has limits, including lack of a sink condition and the inability to predict the fraction of drugs that is absorbed via the lymphatic system and transported by efflux [19]. Attempts have been made to combine artificial membranes or a cell-based permeation step with the current in vitro digestion model [107,136,152], but they may not be able to remove enough drug to mimic effective in vivo drug absorption. Another approach is the use of models that incorporate a means to accurately monitor in vitro lipolysis and to simultaneously assess lipids and drug absorption in rats, as described by Crum et al. [128]. In doing so, they allow for real-time observation of the SNEDDSs lipolysis and drug absorption. However, as in most of in vitro lipolysis models, the experimentations are carried out under conditions not reflecting rat GI fluids but humans or large animals (i.e., dogs). To overcome these conditions, efforts were underway to develop a suitable in vitro rat model of digestion that accurately simulates the composition of rat GI fluids [358]. 

Although the majority of in vitro digestion tests have been carried out under fasted conditions, it has been recognized that these tests should also include fed conditions, with an additional step mimicking gastric lipolysis. One study was conducted to develop a model that initially mimics gastric digestion, then immediately followed by intestinal conditions simulation. The study was conducted under both fasted state and fed state conditions, wherein, after gastric digestion, the experiment was continued after addition of simulated intestinal fluid [133]. 

Considerable efforts are currently underway to generate interesting information that can be used to further refine available in vitro tests in order to design robust models for SNEDDSs evaluation.

## 10. SNEDDSs from an Industrial Perspective

SNEDDSs are initially developed in the lab, and there is a considerable gap between lab and large-scale production. SNEDDSs developments at the lab-scale are well documented. However, literature research revealed that little attention has been paid to challenges related to the large-scale production. This outcome could be explained by several reasons, including the limited experiments, the insufficient information on the large-scale process, the lack of experience and capabilities to cover all the manufacturing processes, or bias towards academic publication [359,360]. The success of any formulation, including SNEDDSs, relies on the bench to large-scale translation. Such translation, however, still possesses serious hurdles related to product stability and batch-to-batch variations that can significantly modify the formulation characteristics, which ultimately impact the therapeutic outcome [360]. However, large-scale production should be conducted in a manufacturing environment that meets Good Manufacturing Practice (GMP) requirements, which rely on strict and robust protocols, validated technical facilities, and well-trained personnel. GMP implementation requires a significant financial support to build a qualified facility.

From the safety point of view, the regulatory status with regard to the toxicity of components is important for the development of a marketed product [27,361]. It should be noted that all excipients are not inert, certain may be toxic, especially at high amounts. SNEDDS components must be chosen from the listed oils, surfactants, and cosolvents provided by the FDA (GRAS excipients). Moreover, the FDA updates the list of excipients in the database quarterly regarding those that are newly approved and incorporated in marketed products, referred to as the Inactive Ingredient Guide (IIG), which are approved and can be added in marketed products. Both IIG and GRAS data can be used by industry as an aid in the development of SNEDDSs formulations [361]. 

The regulatory landscape for SNEDDSs marks a considerable change in formulation approach, moving from the empirical-based formulation method to a more logical formulation approach, such as the QbD approach. The QbD boost in the pharmaceutical industry has been widely recognized and subsequently imbibed due to the guidelines provided by the FDA, EMA, and ICH. QbD, a regulatory-driven approach, aims to build quality from the first design stages with predefined goals by controlling and understanding processes, on basis of a solid science and quality risk management [66]. By doing so, it improves manufacturing processes and ensures the final product quality. Furthermore, this approach saves cost and simplifies production process through the implementation of product quality specifications related to clinical performance, preventing of dose variability as well as improving process design, manufacturing efficiency and post-approval change facility [57].

**Figure 9:**
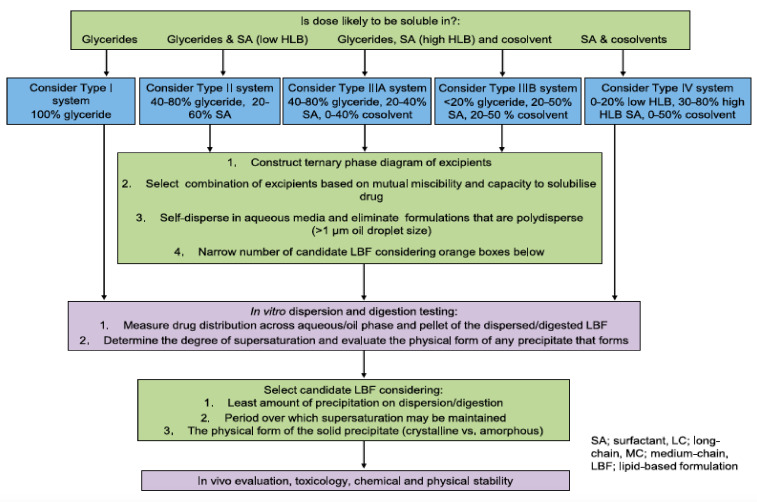
A flowchart providing a general guide to lipid-based formulation design. From [362].

From the formulation point of view, Williams et al. [362] suggested a flowchart that provides a decision tree to formulate a lipid-based drug-delivery system (Figure 9).

This flowchart was developed as part of consortium efforts to rationalize the formulation of lipid-based drug-delivery systems and to elucidate the fate of a drug after its administration via a lipid-based formulation. However, this flowchart provides an academic view of the formulation; thus, a flow diagram representing industrial view is still needed in the public domain.

According to the US FDA, two formulations are therapeutic equivalents if they are pharmaceutical equivalents and can be expected to have the same therapeutic effect and toxicity profile after administration under specified conditions [363]. Two approved drug products are considered therapeutic equivalents if they are pharmaceutical equivalents for which bioequivalence has been demonstrated, and they can be expected to have the same clinical effect and safety profile when administered to patients under the conditions specified in the labeling [363]. Many drug applications for lipid-based formulations have been based on the drug approval in the conventional dosage form given by the same route of administration [364]. Since both tested formulations should contain the same active molecule, comparison of products should be based on single-dose pharmacokinetics and mass balance profile studies [364]. Data obtained from these evaluations will help in determining the dosing regimen for the new formulation. Therefore, regulatory agencies recommend dose-proportionality and multiple-dose studies for the investigated formulation. Additional investigations such as drug interaction studies and studies in special population category may be required to refine the dosing regimen of the product [365]. Currently, there are no specific requirements in place within the FDA or EMA for the preclinical and clinical evaluation of lipid-based formulations in general and SNEDDSs in particular [366,367]. Only reflection articles providing guidelines on the pharmaceutical drug development of a specific type of formulation are found in the literature [368,369,370], and the approval process is essentially the same as that for any other regulated drug device or biologic [367]. 

A review of the literature revealed many SNEDDSs formulations that were approved (EMA and FDA) for the oral delivery of different drugs (Table 5). 

Some of the approved SNEDDSs have been further discontinued. According to the Federal Register, none have been discontinued for efficacy or safety issues. Fortovase^®^ (Saquinavir) was discontinued because a new tablet dosage form with a comparatively low pill burden has been introduced. Kaletra^®^ (Lopinavir/ritonavir lopinavir) was discontinued and replaced by a stable solid dispersion formulation that had higher drug loading, low pill burden, and did not require refrigeration. Agenerase^®^ (Amprenavir) was replaced by a pro-drug (fosamprenavir). The successful marketed products illustrate how SNEDDSs can pass clinical evaluation and result in products providing better care for patients.

## 11. Conclusions

Drug discovery programs provided many new chemical species that are poorly water-soluble. The use of lipid-based formulations in general and SNEDDSs in particular shows great potential in enhancing aqueous solubility, stability, oral absorption and in minimizing inter/intra-patient dose variability. SNEDDSs improve the absorption of drugs by several pathways, including increasing membrane fluidity, bypassing the first-pass effect, and inhibition of P-gp efflux. As described in Figure 2, after SNEDDSs dispersion in the GI tract, nano-emulsions are formed, which facilitate oil hydrolysis by lipases on the oil–water interface. Following this process, micelles along with other colloidal structures made of phospholipids, bile salt, and triglycerides are formed, which increase the transport of the drug through the intestinal barrier. The submicron size of the system with enhanced surface activity allows more robust drug transport through the GI boundary layer, ultimately resulting in better drug absorption and a rapid onset of action.

Previously, SNEDDSs formulations were used to overcome issues related to low aqueous solubility and oral bio-availability drugs. However, the scope of SNEDDSs is far beyond the solubility and dissolution issues. Presently, they have evolved into mucus-permeating, supersaturated, solid and targeted SNEDDSs to tackle issues related to classical SNEDDSs and to make new changes for several applications. Many anti-cancer, anti-diabetic, and anti-viral drug solubility, stability, and bio-availability characteristics were improved via SNEDDSs formulations.

Despite the above-mentioned advancements and modifications in SNEDDSs, there are still areas that need to be addressed to make SNEDDSs commercially attractive. The priority of future research should be based on the mechanisms of action of different SNEDDSs formulations and pharmacokinetic studies, especially on human subjects.

## Figures and Tables

**Table 1:** Commonly used oils, surfactants, and cosolvents.

General Class	Example	Molecular Structure	Commercial Name	Acceptability
**OILS**				
Medium-chain	Triglycerides of capric/caprylic acids	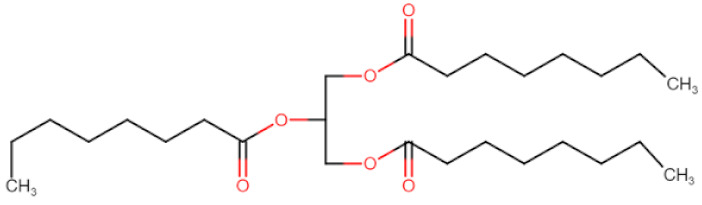	Captex^®^ 300, 350, Labrafac^®^ CC, Crodamol GTCC	P/O/T/Oc/M
	Di-glycerides of capric/caprylic acids	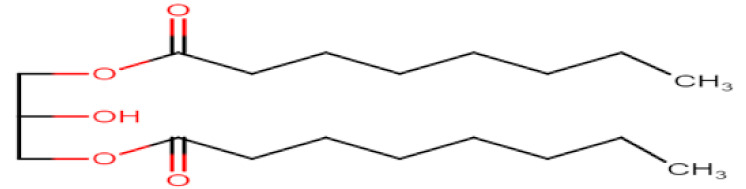	Capmul^®^ MCM, Akoline^®^ MCM	O/T
	Monoglycerides of capric/caprylic acids	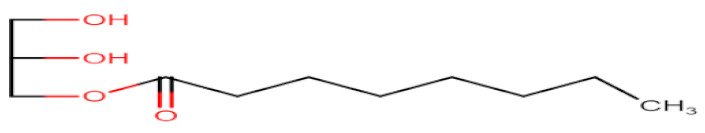	Capryol^®^ 90, Capryol^®^ PGMC, Imwitor^®^ 742	O/T
Long-chain	Glyceryl monooleate	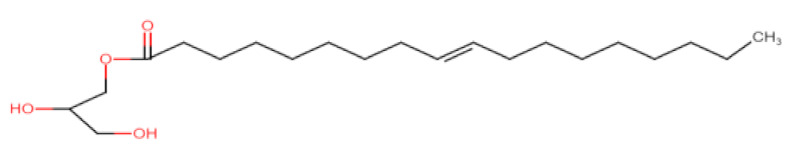	Peceol^®^, Capmul^®^-GMO	O/T
	Glyceryl monolinoleate	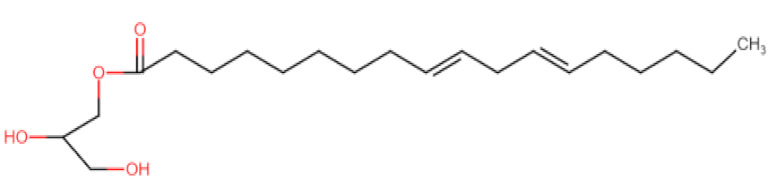	Maisine^®^-35	O/T
Propylene glycol fatty acid esters	Propylene glycol monocaprylate	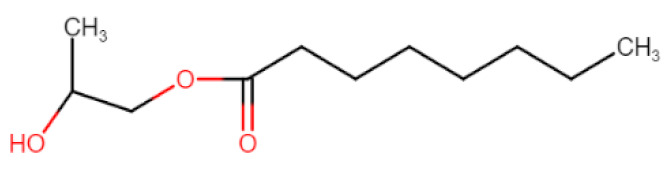	Capmul^®^ PG-8, Sefsol 218	O/T
	Propylene glycol dicaprylate/caprate	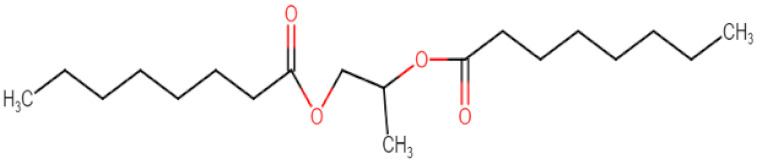	Miglyol^®^ 840, Captex^®^ 200	O/T
	Propylene glycol Monolaurate	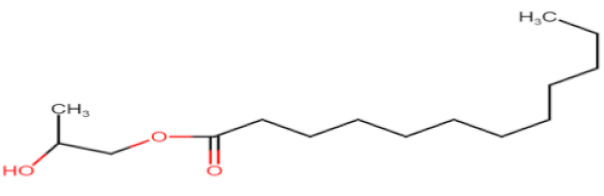	Lauroglycol^®^ 90, Capmul^®^ PG-12, Lauroglycol^®^ FCC	O/T
**SURFACTANTS**				
Polysorbates	Polysorbate esters	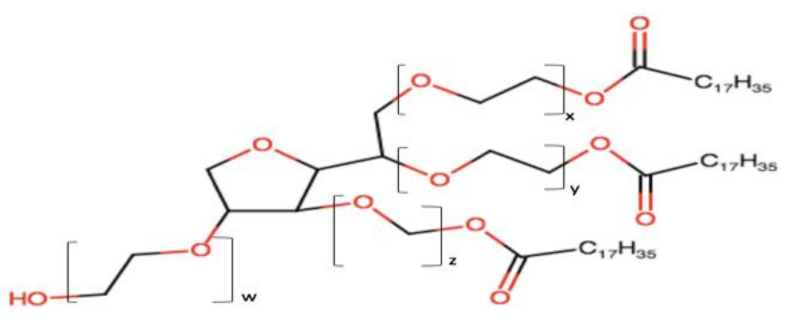	Tween^®^ 20, Tween^®^ 80	P/O/T/Oc M
Sorban esters	Sorban esters	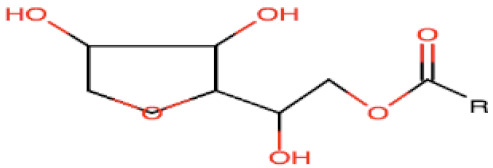	Span^®^ 20,80, Crill^®^ 4	P/O/T/Oc M
Castor oil esters	Ethoxylated castor oil	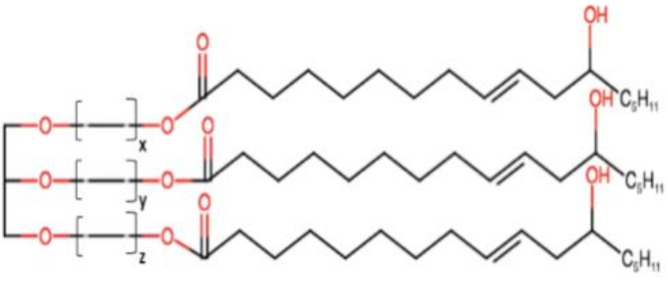	Cremophor^®^-EL, Etocas^®^ 35 HV	O/T
	Hydrogenated castor oil	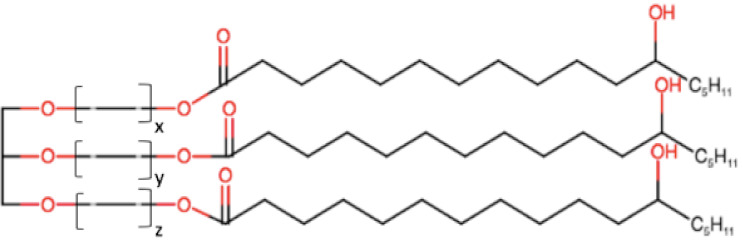	Cremophor^®^ RH40, 60, Croduret^®^ 40	O/T
Polyglycolyzed glycerides	Linoleoyl/Oleoyl Macrogol glycerides	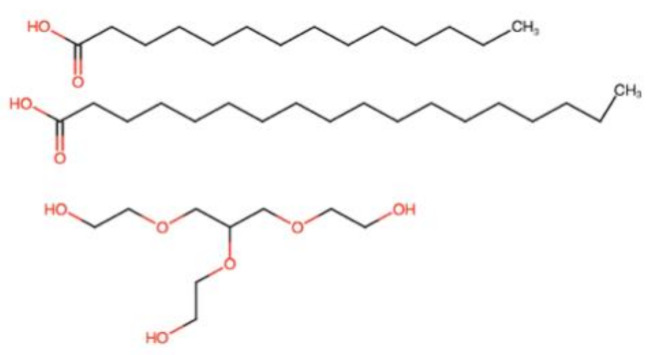	Labrafil^®^ 1944, 2121 CS	O/T
	Caprylocaproyl macrogol glycerides	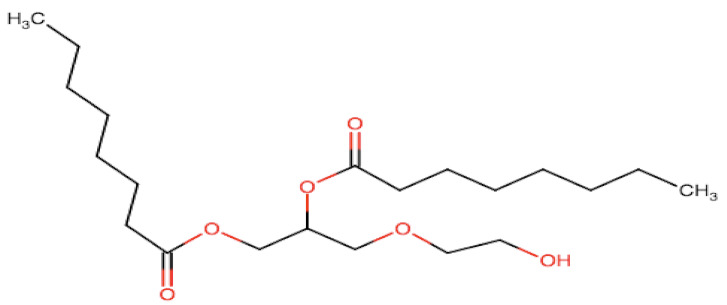	Labrasol^®^	O/T
**COSOLVENTS**				
Alcohols	Short chain Alcohols	R-OH	Ethanol, benzyl alcohol	P/T/Oc/M
	Alkane diols	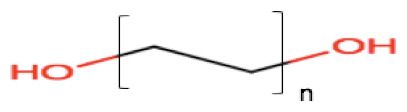	Propylene glycol	P/T/Oc/M
Polyethylene glycols	Polyethylene glycols	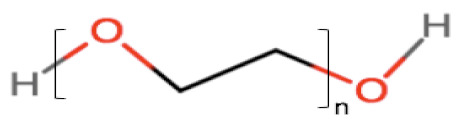	PEG 400, 600	P/T/Oc/M
Esters	Glycerol esters	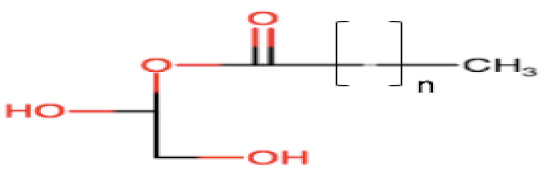	Transcutol^®^	O/T

M: Mucosal; P: Parenteral; O: Oral; Oc: Ocular; T: Topical. *Adapted from* [17].

**Table 2:** The general methods and models used to evaluate SNEDDSs.

	Method/Model	Information Provided
	DLS	Droplet size, PDI, thermodynamic stability
Physico-chemical characterization	Electrophoretic velocimetry	Zeta potential
	Spectrophotometry	Transmittance percentage, cloud point, thermodynamic stability
	TEM, SEM	Morphology, droplet size
	Viscosimeter	Viscosity, thermodynamic stability
	Dissolution apparatus	Drug dissolution, emulsification time
Preclinical in vitro and ex vivo evaluation	pH-stat unit	Formulation digestion, drug distribution across aqueous/oil phase
	PAMPA	Permeation across intestinal barrier
	SPIP	Permeation across intestinal barrier
	IRP	Permeation across intestinal barrier
	CaCO-2	Permeation across intestinal barrier, cytotoxicity
Preclinical In vivo evaluation	Animals	Pharmacokinetic, toxicity, pharmacodynamic
Clinical trials	Humans	Pharmacokinetic, bioequivalence toxicity, pharmacodynamic

PAMPA: parallel artificial membrane permeability assay, SPIP: single-pass intestinal perfusion, IRP: intestinal recirculating perfusion.

**Table 3:** Examples of preclinical studies reporting enhanced dissolution and bio-availability of drugs upon their incorporation into SNEDDSs.

Class	Drug	Components	In Vitro/Vivo Observation	References
**ANTI-CANCER**	Docetaxel	Capryol^®^ 90, Labrasol^®^, Transcutol^®^ HP	AUC_0-t_ and C_max_ increased 6.4 and 6.5-fold, respectively compared to docetaxel aqueous solution.	[196]
	Erlotinib	Labrafil^®^ M2125 CS, Labrasol^®^, Transcutol^®^ HP, Aerosil^®^ 200, Dextran 40	AUC_0–t_ and C_max_ increased 2.1 and 2.4-fold, respectively in case of dextran-based solid SEDDS compared to erlotinib powder.	[197]
	Paclitaxel	Sesame oil, Labrasol^®^, Sodium deoxycholate	AUC_0–t_ and C_max_ increased to 2.7 and 3.99-fold, respectively compared to drug suspension.	[194]
	Lycopene	LCT, Tween^®^ 85, Cremophor^®^ RH, Gelucire^®^	AUC_0–t_ and C_max_ increased 2.3 and 2.85-fold, respectively compared to Lycovit^®^.	[198]
	Methotrexate	Ethyl oleate, Tween^®^ 80, Propylene glycol	AUC_0–24_ and C_max_ increased 1.57 and 1.68-fold, respectively compared to native drug.	[199]
	Irinotecan	Capmul^®^ CM-C8, Cremophor^®^ EL, Pluronic L-121	AUC_0–t_ and C_max_ increased 4.2 and 1.7-fold, respectively compared to drug suspension.	[200]
**CARDIOVASCULAR AND ANTI-HYPERTENSIVE**	Carvedilol	Labrafil^®^ M1944CS, Tween^®^ 80, Transcutol^®^	Relative bio-availability enhanced by 4.1 times compared with tablet.	[201]
	Felodipine	Miglyol^®^ 812, Cremophor^®^ RH 40, Tween^®^ 80, Transcutol^®^ HP, Silicon dioxide	AUC_0–t_ increased 2-fold compared to conventional tablets.	[202]
	Clinidipine	Capryol^®^ 90, Tween^®^ 80, Transcutol^®^	The absorption of the drug was enhanced from liquid-SEDDS as 99 % of the drug was transported from mucosal to serosal side of the rat intestine within 90 min from SEDDS in comparison to only 42.2% from that of the pure drug suspension.	[203]
	Valsartan	Triacetin or Castor oil, Tween^®^ 80, PEG 600	For triacetin-SNEDDS 5 and 2.4-fold increase in Cmax and AUC, respectively; for castor oil SNEDDS 8 and 3.6-fold increase in Cmax and AUC, respectively.	[204]
	Rosuvastatin	Peceol^®^, Tween^®^ 80, Transcutol^®^ HP	In vivo pharmacokinetic studies revealed 1.8 and 5.7-fold enhancement in AUC_0-t_ and C_max_, respectively, and 0.33-fold reduction in T_max_ of drug from the SNEDDS vis-à-vis the pure drug suspension.	[173]
	Atenolol	Tartaric acid, Captex^®^, Span^®^ 80, Oleic acid	*Ex vivo* intestinal permeability studies revealed that atenolol SDEDDS exhibited better drug permeation compared to atenolol or atenolol-tartaric acid suspension.	[205]
	Ramipril	Sefsol, Tween^®^ 80, Carbitol	2.29-fold improvement in oral bio-availability compared with free drug suspension.	[104]
**ANTI-DIABETIC**	Insulin	Miglyol^®^, Cremophor^®^ RH40, MCM C-10, Ethanol	AUC_0–t_ increased 2.7-fold compared to insulin solution.	[206]
	Glibenclamide	Cotton oil, Tween^®^ 80, Propylene glycol	AUC_0–t_ increased 1.4-fold compared to free drug.	[207]
	Trans-cinnamic acid	Isopropyl myristate, Cremophor^®^ EL, PEG 400	The efficacy of trans-cinnamic acid in both hyperglycemia and glucolipid metabolic disorder was enhanced in SNEDDS compared to the drug suspension.	[208]
	Gliclazide	Capryol^®^ 90, Cremophor^®^ EL, Akoline^®^ MCM	Enhancement in oral bio-availability as compared to the free drug.	[209]
	Exenatide	Cremophor^®^ EL, Labrafil^®^ 1944, Capmul^®^-PG 8, propylene glycol	14.6-fold higher relative bio-availability versus subcutaneous exenatide solution.	[210]
**ANTIOXIDANT**	Quercetin	Capmul^®^, Tween^®^ 20, Ethanol	23.7-fold increase in the cell uptake of quercetin when incorporated in SEDDS compared to free drug.	[211]
	Resveratrol	Miglyol^®^ 812, Montanox, Labrasol^®^, Gelucire^®^, Ethanol	The absorptive fluxes through the intestinal epithelium from the nano-emulsions were significantly increased compared to an ethanolic control solution.	[212]
	Genistein	Labrafac^®^ lipophile 1349, Maisine^®^-35, Cremophor^®^ EL, Labrasol^®^, Transcutol^®^	95% of drug release in 5 min.	[213]
	Retinol acetate	Soybean oil, Capmul^®^, Cremophor^®^ EL	Improved in dissolution rate.	[214]
	Coenzyme Q10	Lauroglycol^®^ FCC, Witepsol^®^ H335, Solutol^®^ HS 15	5-fold improvement in oral bio-availability compared to free drug.	[39]
**ANTI-VIRAL,** **ANTI-BACTERIAL, ANTI-FUNGAL, AND ANTIPROTOZOAL**	Darunavir	Lauroglycol^®^ 90, Tween^®^ 80, Transcutol^®^ HP	Enhancement in AUC_0-t_, oral bio-availability and C_max_, 1.45,5.8 and 7.5-fold, respectively compared to free drug.	[215]
	Nelfinavir mesylate	Maisine^®^ 35-1, Tween^®^ 80, Transcutol^®^ HP	4.5-fold improvement in permeability and 3.6-fold improvement in bio-availability.	[113]
	Lopinavir	Maisine^®^, Tween^®^-80, Transcutol^®^ HP	Enhanced oral bio-availability (3.9-fold) compared to the pure drug.	[216]
	Acyclovir	Sunflower oil, Tween^®^ 60, Glycerol	3.5-fold increase in oral bio-availability compared to the pure drug suspension.	[217]
	Rifampicin	Capmul^®^ MCM C, Cremophor^®^-EL, Labrasol^®^	3.72 and 5.22-fold improvement in AUC_0–t_ and C_max_, respectively compared to drug suspension.	[218]
	Amphotericin B	Peceol^®^, PEG-200, Distearoylphos-phatidylethanolamine	Amphotericin B-SEDD treatment significantly decreases total fungal colony forming unit concentrations compared to non-treated controls without significant changes in plasma creatinine levels in the A. fumigatus infected rats.	[219]
	Satranidazole	Oleic acid, Tween^®^ 20, PEG 400	SNEDDSs formulations showed a drug release of greater than 70% in 45 minutes whereas marketed preparation showed more than 70% of drug release in 90 min.	[220]

**Table 4:** Pharmacokinetics data reporting enhanced bio-availability from Self-Emulsifying Drug Delivery Systems (SEDDS) in human subjects.

Drug	Components	In Vivo Observation	References
Vitamin E	Palm oil, Tween^®^ 80, Span^®^ 80	3-fold higher oral bio-availability from SEDDSs.	[223]
Cyclosporin	Corn oil glycerides, Cremophor^®^ RH40, PG, DL-α-tocopherol and ethanol	AUC_0–t_ and C_max_ increased 1.18 and 1.17-fold, respectively from SEDDSs.	[224]
Tocotrienols	Tocomin, Soybean oil Tween^®^ 80 Labrasol^®^	2 to 3-fold higher oral bio-availability from SEDDSs.	[82]
Saquinavir (Fortovase^®^)	Medium-chain mono- and di-glycerides	Increased oral bio-availability up to 331% from Fortovase^®^ compared to Invirase^®^.	[198]
Simvastatin	Labrafil^®^, Tween^®^ 80, Transcutol^®^ HP	1.55 and 1.5 increased in Cmax and AUC_0–t_, respectively from SNEDDSs.	[225]
Vitamin K	Vitamin K, Labrasol^®^, Transcutol^®^ HP	Enhancement in vitamin K relative bio-availability from SNEDDSs.	[222]

**Table 5:** Non-exhaustive list of marketed SNEDDSs for oral administration.

Drug Name	Trade Name (Company)	Composition	Dosage Form
Ritonavir	Norvir^®^ Abbott Laboratories	Ole Oleic acid, Cremophor^®^-EL, ethanol, butylated hydroxytoluene	Soft capsules
Tipranavir	Aptivus^®^ (Boehringer Ingelheim)	Mono/di-glycerides of caprylic acids, Cremophor^®^ EL ethanol, propylene glycol	Soft capsules
Cyclosporine	Sandimmune^®^ (Novartis)	Corn oil/olive oil, Labrafil^®^ M 1944 CS, ethanol, α-tocopherol	Soft capsule
	Neoral^®^ (Novartis)	Mono-, di- and triglycerides of corn oil, Cremophor^®^ RH40, propylene glycol, ethanol, D-α-tocopherol	Oral solution and soft capsules
Isotretinoin	Accutane^®^ (Roche)	Beeswax, hydrogenated soybean oil flakes, hydrogenated vegetable oil, soybean oil Olive, polyoxyethylated oleic glycerides, ethanol	Soft capsules
Sirolymus	Rapamune^®^ (Wyeth-Ayerst)	Phosphatidylcholine, mono- and di-glycerides, soy fatty acids, Tween^®^ 80, ethanol, propylene glycol, ascorbyl palmitate	Oral solution
